# Comparative efficacy and safety of adjuvant nivolumab versus other treatments in adults with resected melanoma: a systematic literature review and network meta-analysis

**DOI:** 10.1186/s12885-020-07538-1

**Published:** 2021-01-05

**Authors:** Kabirraaj Toor, Mark R. Middleton, Keith Chan, Adenike Amadi, Andriy Moshyk, Srividya Kotapati

**Affiliations:** 1 Precision HEOR, 1505 West 2nd Avenue, Vancouver, BC V6H 3Y4 Canada; 2 University of Oxford, Old Road Campus Research Building, Roosevelt Drive, Oxford, OX3 7DQ UK; 3 Bristol Myers Squibb, Unit 2 Uxbridge Business Park, Uxbridge, UB8 1DH UK; 4 Bristol Myers Squibb, Route 206 and Province Line Road, Princeton, NJ 08543 USA

**Keywords:** Adjuvant treatment, Systematic literature review, Network meta-analysis, Nivolumab, Efficacy, Safety

## Abstract

**Background:**

Immune checkpoint inhibitors and targeted therapies are approved for adjuvant treatment of patients with resected melanoma; however, they have not been compared in randomized controlled trials (RCTs). We compared the efficacy and safety of adjuvant nivolumab with other approved treatments using available evidence from RCTs in a Bayesian network meta-analysis (NMA).

**Methods:**

A systematic literature review was conducted through May 2019 to identify relevant RCTs evaluating approved adjuvant treatments. Outcomes of interest included recurrence-free survival (RFS)/disease-free survival (DFS), distant metastasis-free survival (DMFS), all-cause grade 3/4 adverse events (AEs), discontinuations, and discontinuations due to AEs. Time-to-event outcomes (RFS/DFS and DMFS) were analyzed both assuming that hazard ratios (HRs) are constant over time and that they vary.

**Results:**

Of 26 identified RCTs, 19 were included in the NMA following a feasibility assessment. Based on HRs for RFS/DFS, the risk of recurrence with nivolumab was similar to that of pembrolizumab and lower than that of ipilimumab 3 mg/kg, ipilimumab 10 mg/kg, or interferon. Risk of recurrence with nivolumab was similar to that of dabrafenib plus trametinib at 12 months, however, was lower beyond 12 months (HR [95% credible interval] at 24 months, 0.46 [0.27–0.78]; at 36 months, 0.28 [0.14–0.59]). Based on HRs for DMFS, the risk of developing distant metastases was lower with nivolumab than with ipilimumab 10 mg/kg or interferon and was similar to dabrafenib plus trametinib.

**Conclusion:**

Adjuvant therapy with nivolumab provides an effective treatment option with a promising risk–benefit profile.

**Supplementary information:**

**Supplementary information** accompanies this paper at 10.1186/s12885-020-07538-1.

## Background

The incidence of melanoma, a type of skin cancer that develops from melanocytes, has consistently increased worldwide [1, 2]. In 2009, more than 850,000 people in the United States had a history of melanoma [2, 3]. Melanoma is surgically treated with curative intent if diagnosed at an early stage; however, once melanoma metastasizes, treatment with curative intent is not an option [1]. In patients with resected melanoma, clinically important outcomes include recurrence-free survival (RFS)/disease-free survival (DFS), which measures the curative efficacy of treatment, and distant metastasis-free survival (DMFS), which measures the curative efficacy of treatment in preventing advanced or metastatic disease [4].

Traditionally, patients with intermediate-risk (stages II and IIIA) and high-risk (stage IIIB, stage IIIC, and resectable stage IV) melanoma have been treated with regional radiotherapy and immunostimulants, in addition to watchful waiting after surgery (adjuvant setting) [2, 5, 6]. Without active treatment, only 55 to 60% of patients remain recurrence-free at 1 year [7–9]. Since 2011, adjuvant treatment options have expanded to include biologic agents such as interferon (IFN)-alpha and immune checkpoint inhibitors, such as cytotoxic T-lymphocyte antigen 4 and programmed death (PD)-1 inhibitors [1, 2]. IFN-alpha was the first agent to demonstrate RFS/DFS benefit in a randomized controlled trial (RCT) [2, 10]. Additionally, three immune checkpoint inhibitors (ipilimumab, nivolumab, and pembrolizumab), as well as the BRAF/MEK inhibitor combination dabrafenib plus trametinib, have been approved by the US Food and Drug Administration (FDA) for the adjuvant treatment of patients with melanoma [11–14]. Most of the trials involving these agents utilized placebo rather than an active comparator as the control and reported that 70 to 88% of patients remained recurrence-free 1 year after initiating active adjuvant treatment [7–9]. Although ipilimumab 10 mg/kg and nivolumab 3 mg/kg have been compared head to head in the phase III CheckMate 238 trial [15], efficacy and safety comparisons between nivolumab and other adjuvant treatments in clinical trials are lacking.

Network meta-analysis (NMA) is a statistical method that allows indirect comparisons between treatments when head-to-head evidence is not available and naive comparisons would be prone to selection bias and imbalance in a variety of baseline characteristics. Specifically, NMA can be used to combine direct and indirect evidence for any interventions that form a network of RCTs in which each trial has at least one intervention (active or placebo) in common with another trial and all trials are sufficiently similar [16, 17]. Furthermore, NMA is based on the analysis of relative treatment effects rather than a comparison of absolute values of efficacy outcomes. Two NMAs on adjuvant treatment options in melanoma were published recently [18, 19]. One of these studies reported treatment effects with an assumption of proportional hazards (i.e., a constant hazard ratio [HR]) in comparing the safety of dabrafenib plus trametinib with other adjuvant therapies, including nivolumab [18]. The other study allowed for time-varying treatment effect when comparing pembrolizumab with adjuvant therapies other than nivolumab and did not present a safety analysis [19]. The current study was conducted using publicly available evidence from RCTs identified through a systematic literature review (SLR), which was then synthesized by means of an NMA to assess the efficacy and safety of nivolumab versus other treatment options in patients with resected melanoma by using both constant and time-varying treatment-effect assumptions.

## Methods

### Literature search

Our SLR included RCTs that reported the comparative efficacy and safety of pharmacologic interventions for the adjuvant treatment of patients with resected melanoma in terms of RFS/DFS, DMFS, all-cause grade 3/4 adverse events (AEs), discontinuations, and discontinuations due to AEs. Studies in patients with stage III/IV melanoma published through May 2019 were included; studies assessing patients with stage II melanoma were also included if this population was assessed in addition to the stage III/IV population. To assess the impact of studies assessing stage II melanoma patients, a subgroup (sensitivity) analysis was conducted in studies that included only patients with resectable stage III/IV melanoma or reported stage III/IV subgroup data. The quality of individual trials was assessed using the Cochrane risk-of-bias tool. Full details and results of the SLR are provided in Additional file 1: Appendix A.

A feasibility assessment was conducted to gauge the appropriateness of proceeding with an NMA. This process included the following steps: (1) determination of whether the RCT formed a single evidence network for each outcome of interest (RFS/DFS, DMFS, grade 3/4 AEs, discontinuations, and discontinuations due to AEs) and (2) assessment of the distribution of treatment, outcomes, study, and patient characteristics that affected treatment effects across direct comparisons of the evidence networks when head-to-head evidence existed. Network meta-analyses were conducted in a Bayesian framework for the RCTs identified in the SLR that formed part of a single evidence network and were deemed sufficiently similar for each population and outcome of interest. Consistency between direct and indirect estimates was evaluated for closed loops (see Additional file 2: Appendix B for results of consistency checks). For binary outcomes, such as grade 3/4 AEs, discontinuations, and discontinuations due to AEs, NMAs were performed based on the proportion of patients experiencing the event of interest using a regression model with a binomial likelihood and logit link.

For survival outcomes, specifically RFS/DFS and DMFS (reported either as HRs or Kaplan–Meier [KM] curves), NMAs were conducted based either on the assumption that HRs remained constant over time or that HRs varied over time. In a typical survival analysis, the ratio of the risk of an event occurring between two treatments was assumed to be constant over time (ie, proportional hazards assumption); however, this assumption did not hold for some comparative survival analyses, evidenced by overlapping KM curves or based on the results of the Grambsch and Therneau test. In such cases, an additional analysis that allowed for time-varying HRs was conducted using methods described previously [20, 21]. For these methods, the hazard functions of the interventions in a trial were modeled using known survival functions (such as Weibull or Gompertz, generally referred to as fractional polynomials), and differences in the parameters were considered in the multidimensional treatment effects, which were synthesized and indirectly compared across studies in the NMA. Because of this approach, treatment effects were represented by multiple parameters, rather than a single parameter. In this study, the model introduced by Jansen [20] was used for NMAs of RFS/DFS and DMFS. Normal non-informative prior distributions were used for all parameters (mean of 0; variance of 10,000). Relative treatment effects were expressed as HRs for RFS/DFS and DMFS and odds ratios for AEs and discontinuations, with 95% credible intervals (CrIs).

## Results

### Evidence base

**Fig. 1:**
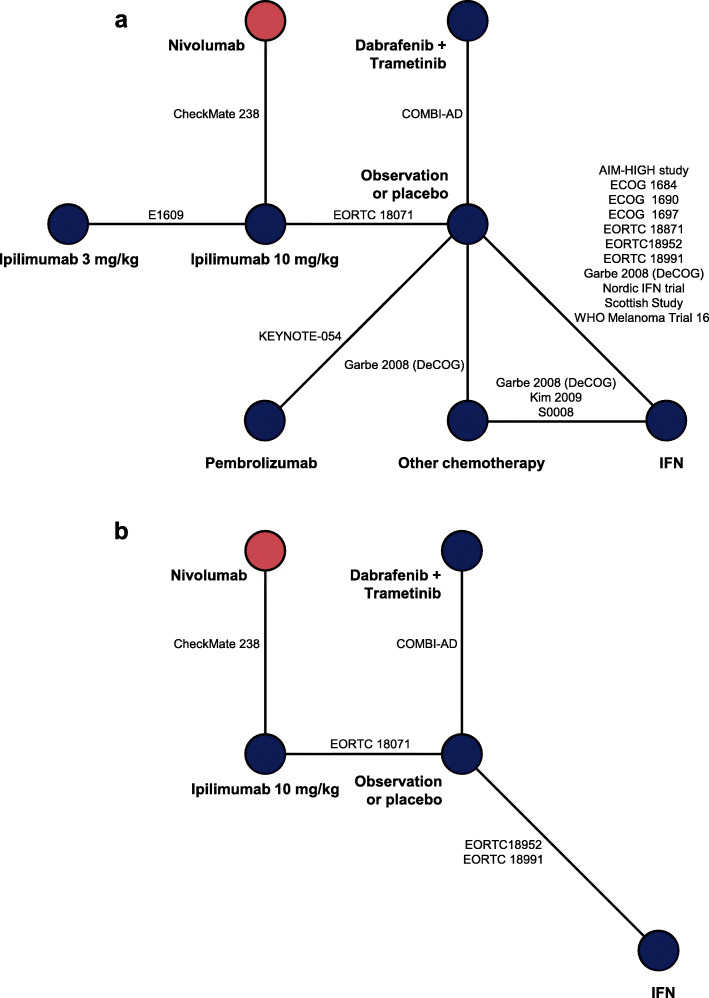
Network diagrams of randomized controlled trials for base-case efficacy outcomes in patients with stage II–IV melanoma for **a** RFS/DFS and **b** DMFS. *DFS* disease-free survival, *DMFS* distant metastasis-free survival, *IFN* interferon, *RFS* recurrence-free survival

An SLR conducted through May 2019 identified 11 new studies in addition to the 41 studies identified in an earlier SLR, generating an evidence base of 52 studies (Additional file 1: Appendix A, Fig. A.1). These studies represent 26 RCTs. A feasibility assessment excluded seven trials, comprising two trials that assessed only patients with mucosal melanoma, two that included patients with stage I disease and did not provide data for the stage III/IV disease subgroup, one that pooled ipilimumab dosing groups, and two that were treated as single-arm trials due to aggregation of nodes (interventions of interest). Overall, 19 trials were included in the NMA. Among these trials, IFN was the most frequently assessed treatment (*n* = 13), followed by other chemotherapy (*n* = 4), and ipilimumab 10 mg/kg (*n* = 3). Ipilimumab 3 mg/kg, nivolumab, pembrolizumab, and dabrafenib plus trametinib were assessed in one trial each. The analysis of RFS/DFS included 18 trials assessing eight treatments (Fig. 1a), and the analysis of DMFS included five trials assessing five treatments (Fig. 1b).

Characteristics of the 26 RCTs included in the feasibility assessment for the NMA are provided in Table 1 [7, 10, 15, 22–47]. The treatments included in this analysis were consistent across the network because the eligibility criteria for the interventions were restricted primarily to FDA-approved doses. Although ipilimumab 3 mg/kg and 10 mg/kg were used for the treatment of patients with resected melanoma in the studies included in this NMA, only the 10-mg/kg dose is approved in the adjuvant setting. The observation/placebo and other-chemotherapy nodes consisted of more than one treatment type. Among the 19 trials included in the observation/placebo node, observation was the control in 16 trials and placebo served as the control in three trials. In the other-chemotherapy node, four of the five chemotherapies were either dacarbazine or IFN; however, the treatment regimens were different.

**Table 1:** Characteristics of randomized controlled trials included in the NMA

Trial	Primary publication	ClinicalTrials.gov identifier	Treatment 1	Treatment 2	Treatment 3	Blinding	Phase	Multicenter	Region
*Trials included in final NMAs*									
ECOG 1697 [22]	Agarwala et al. (2017)	NCT00003641	IFN alfa-2b (high-dose)	Observation	NA	Open-label	III	Yes	Australia, Canada, South Africa, USA
Scottish study [23]	Cameron et al. (2001)	NA	IFN alfa-2b (low-dose)	Observation	NA	Open-label	NR	Yes	Scotland
WHO Melanoma Programme Trial 16 [24]	Cascinelli et al. (2001)	NA	IFN alfa-2b (low-dose)	Observation	NA	Open-label	NR	Yes	Italy
EORTC 18071 [25]	Eggermont et al. (2015)	NCT00636168	Ipilimumab	Placebo	NA	Double-blind	III	Yes	Australia, Austria, Belgium, Canada, Czech Republic, Denmark, Finland, France, Germany, Italy, Netherlands, Norway, Poland, Russia, Spain, Sweden, Switzerland, United Kingdom, USA
EORTC 18952 [26]	Eggermont et al. (2005)	NA	IFN alfa-2b (high-dose/ 13 months)	IFN alfa-2b (low-dose/ 25 months)	Observation	Open-label	III	Yes	Austria, Belgium, Bulgaria, Croatia, Estonia, Finland, France, Germany, Hungary, Israel, Italy, Poland, Portugal, Russia, Serbia and Montenegro, Slovakia, Spain, Sweden, Switzerland, Netherlands, Turkey, United Kingdom
EORTC 18991 [27]	Eggermont et al. (2008)	NCT00006249	PEG IFN alfa-2b	Observation	NA	Open-label	III	Yes	Australia, Belgium, Bulgaria, Croatia, Czech Republic, Estonia, France, Germany, Israel, Italy, Netherlands, Poland, Portugal, Slovenia, Spain, Switzerland, Turkey, United Kingdom
KEYNOTE-054 [28, 29]	Eggermont et al. (2018)	NCT02362594	Pembrolizumab	Placebo	NA	Double-blind	III	Yes	Australia, Belgium, Canada, Denmark, Finland, France, Germany, Israel, Italy, Japan, Netherlands, New Zealand, Norway, Poland, Portugal, Russian Federation, Serbia, Spain, Sweden, Switzerland, United Kingdom, USA
S0008 [30]	Flaherty et al. (2014)	NCT00006237	IFN alfa-2b (high-dose)	Other chemotherapy	NA	Open-label	III	Yes	Australia, USA
Garbe 2008 (DeCOG) [31]	Garbe et al. (2008)	NA	IFN alfa-2a (low-dose)	IFN alfa-2a + dacarbazine	Observation	Open-label	III	Yes	Germany, Switzerland
AIM HIGH Study [32]	Hancock et al. (2004)	NA	IFN alfa-2a (low-dose)	Observation	NA	Open-label	III	Yes	United Kingdom
Nordic IFN Trial [33]	Hansson et al. (2011)	NCT01259934	IFN alfa-2b (1 year)	IFN alfa-2b (2 years)	Observation	Open-label	III	Yes	Denmark, Finland, Norway, Sweden
Kim et al. 2009 [34]	–	NA	Cisplatin, vinblastine, dacarbazine, IFN alfa-2b, interleukin-2	IFN alfa-2b (high-dose)	IFN alfa-2b (low-dose)	Open-label	III	NR	USA
ECOG 1690 [35]	Kirkwood et al. (2000)	NA	IFN alfa-2b (low-dose)	IFN alfa-2b (high-dose)	Observation	Open-label	III	Yes	USA
ECOG 1684 [10]	Kirkwood et al. (1996)	NA	IFN alfa-2b (high-dose)	Observation	NA	Open-label	III	Yes	USA
EORTC 18871 [36]	Kleeberg et al. (2004)	NA	rIFN alfa-2b	Observation	NA	Open-label	III	Yes	Austria, Belgium, Czech Republic, Estonia, France, Germany, Great Britain, Greece, Israel, Poland, Spain, Switzerland, Yugoslavia
COMBI-AD [7, 37]	Long et al. (2017)	NCT01682083	Dabrafenib + trametinib	Placebo	NA	Double-blind	III	Yes	Argentina, Australia, Austria, Belgium, Brazil, Canada, Czech Republic, Denmark, France, Germany, Greece, Israel, Italy, Japan, Netherlands, New Zealand, Norway, Poland, Russia, Spain, Sweden, Switzerland, Taiwan, United Kingdom, USA
Stadler et al. (2006) [38]	–	NA	Dacarbazine + natural human IFN alfa	Observation	NA	Open-label	NR	Yes	Germany
E1609 [39]	Tarhini et al. (2017)	NCT01274338	Ipilimumab (high-dose)	Ipilimumab (low-dose)	IFN alfa-2b (high-dose)	Open-label	III	Yes	Canada, USA
CheckMate 238 [15, 40]	Weber et al. (2017)	NA	Nivolumab	Ipilimumab	NA	Double-blind	III	Yes	Argentina, Australia, Austria, Belgium, Canada, Czech Republic, Finland, France, Greece, Hungary, Ireland, Israel, Italy, Japan, Korea, Netherlands, Norway, Poland, Romania, South Africa, Spain, Sweden, Switzerland, Taiwan, United Kingdom, USA
*Trials excluded from final NMAs*									
Eigentler et al. 2016 (DeCOG) [41]	Eigentler et al. (2016)	NCT00204529	PEG IFN alfa-2a	IFN alfa-2a (low-dose)	NA	Open-label	III	Yes	Austria, Germany
EADO study [42]	Grob et al. (2013)	NCT00221702	PEG IFN alfa-2b	IFN alfa-2b (low-dose)	NA	Open-label	III	Yes	Austria, France, Germany
Lian et al. (2013) [43]	–	NA	IFN alfa-2b (high-dose)	Chemotherapy	Observation	NR	II	No	China
Sunbelt Melanoma Trial [44]	McMasters et al. (2016)	NA	IFN alfa-2b (high-dose)	Observation	NA	Open-label	III	Yes	Canada, USA
MM-ADJ-5 [45]	Mohr et al. (2015)	NCT00226408	IFN alfa-2b (high-dose)	IFN alfa-2b (intermittent high-dose)	NA	Open-label	III	Yes	Austria, Germany, Greece, Switzerland
Tobin et al. (2018) [46]	–	NCT02403778	Ipilimumab	Ipilimumab plus ATRA	NA	Open-label	II	No	USA
Wang et al. (2015) [47]	–	NA	IFN alfa-2b (high-dose)	Observation	NA	Open-label	II	No	China

*ATRA* all-trans retinoic acid, *IFN* interferon, *NA* not applicable, *NMA* network meta-analysis, *NR* not reported, *PEG* pegylated, *rIFN* recombinant IFN

Baseline patient characteristics were largely similar across the trials, including age and race (Additional file 1: Appendix A, Table A.11). However, differences in patient characteristics were observed in American Joint Committee on Cancer stage and *BRAF* mutation status at baseline. To assess whether differences in patient characteristics had an effect on the analysis, a subgroup analysis was conducted in patients with stage III/IV disease. However, a lack of available data and network connectivity precluded a subgroup analysis based on *BRAF* status, as only three trials reported this information.

### Efficacy

Based on HRs for RFS/DFS, the risk of recurrence was similar between nivolumab and dabrafenib plus trametinib (HR 1.06, CrI 0.77–1.45) and between nivolumab and pembrolizumab (HR 0.92, CrI 0.67–1.29) when the HR was constant over time (Table 2). Risk of recurrence was lower with nivolumab than with ipilimumab 3 mg/kg, ipilimumab 10 mg/kg, or IFN. As the assumption of proportional hazards did not hold, a subsequent analysis was conducted in which HR varied over time. In this analysis, the risk of recurrence with nivolumab was similar to that with dabrafenib plus trametinib at 12 months (HR 1.02, 95% CrI 0.71–1.47), but was lower at later time points (HR at 24 months 0.46, 95% CrI 0.27–0.78; HR at 36 months 0.28, 95% CrI 0.14–0.59) (Table 3).

**Table 2:**
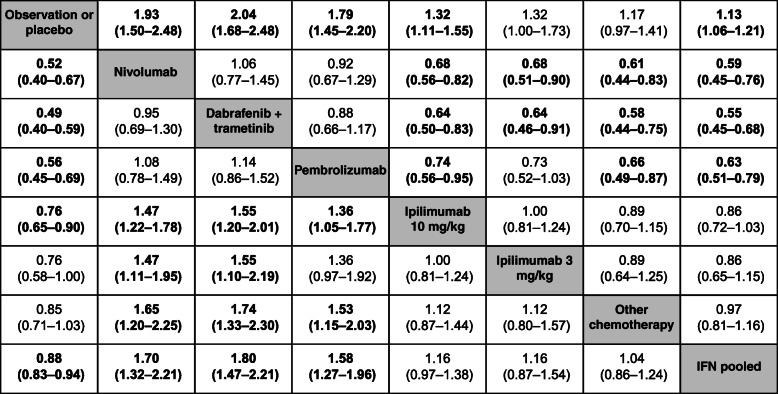
Constant HR estimates^a^ from a fixed-effects NMA of RFS/DFS in patients with resected stage II–IV melanoma

^a^The value in each cell represents the hazard ratio (95% credible interval) for the comparison of the treatment indicated in that row versus the treatment indicated in that column; bolded values are statistically significant at the 0.05 significance level

*DFS* disease-free survival, *HR* hazard ratio, *IFN* interferon, *NMA* network meta-analysis, *RFS* recurrence-free survival

**Table 3:** Time-varying HR estimates^a^ from a fixed-effects NMA of RFS/DFS (p1 = 0, p2 = −1, scale, shape ×1) in patients with resected stage II–IV melanoma

Treatment	Time points			
	12 months	24 months	36 months	48 months
NIVO vs. OBS/PBO	**0.58 (0.43–0.77)**	**0.51 (0.33–0.80)**	**0.46 (0.26–0.86)**	**0.43 (0.21–0.91)**
NIVO vs. DAB+TRAM	1.02 (0.71–1.47)	**0.46 (0.27–0.78)**	**0.28 (0.14–0.59)**	**0.20 (0.08–0.49)**
NIVO vs. PEM	1.19 (0.81–1.76)	1.06 (0.56–2.05)	0.96 (0.40–2.29)	0.88 (0.30–2.51)
NIVO vs. IPI (10 mg/kg)	**0.69 (0.55–0.86)**	**0.66 (0.46–0.96)**	0.65 (0.40–1.07)	0.64 (0.35–1.17)
NIVO vs. IPI (3 mg/kg)	0.75 (0.54–1.04)	0.93 (0.58–1.48)	1.06 (0.57–2.01)	1.16 (0.54–2.55)
NIVO vs. CHEM	**0.63 (0.44–0.89)**	**0.53 (0.32–0.86)**	**0.47 (0.24–0.91)**	**0.42 (0.19–0.95)**
NIVO vs. IFN	**0.64 (0.48–0.86)**	**0.55 (0.36–0.88)**	**0.49 (0.27–0.93)**	**0.46 (0.22–0.97)**

^a^The value in each cell represents the hazard ratio (95% credible interval) for the comparison of the treatments; bolded values are statistically significant at the 0.05 significance level

*CHEM* other chemotherapy, *DAB* dabrafenib, *DFS* disease-free survival, *HR* hazard ratio, *IFN* interferon, *IPI* ipilimumab, *NIVO* nivolumab, *NMA* network meta-analysis, *OBS* observation, *PBO* placebo, *PEM* pembrolizumab, *RFS* recurrence-free survival, *TRAM* trametinib

Based on HRs for DMFS, the risk of developing distant metastases was lower with nivolumab than with ipilimumab 10 mg/kg or IFN but was similar to that of dabrafenib plus trametinib (Table 4). In the analysis with time-varying HR, the risk of developing distant metastases was lower for nivolumab compared with observation/placebo at 12 months (HR 0.67, 95% CrI 0.48–0.93), but was not different at later time points or compared with other treatments (Table 5). Pembrolizumab was not included in the DMFS analysis due to a lack of publicly available data. Results for RFS/DFS and DMFS were consistent between subgroup analyses that included patients with stage III/IV disease and overall analyses that included patients with stage II–IV disease (see Additional file 3: Appendix C).

**Table 4:**
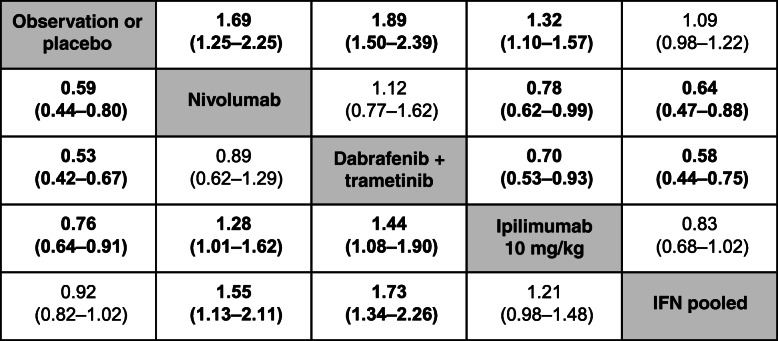
Constant HR estimates^a^ from a fixed-effects NMA of DMFS in patients with stage II–IV melanoma

^a^The value in each cell represents the hazard ratio (95% credible interval) for the comparison of the treatment indicated in that row versus the treatment indicated in that column; bolded values are statistically significant at the 0.05 significance level

*HR* hazard ratio, *DMFS* distant metastasis-free survival, *IFN* interferon, *NMA* network meta-analysis

**Table 5:** Time-varying HR estimates^a^ from a fixed-effects NMA for DMFS (p1 = 0, p2 = −0.5, scale, shape ×1) in patients with stage II–IV melanoma

Treatment	Time points			
	12 months	24 months	36 months	48 months
NIVO vs. OBS/PBO	**0.67 (0.48–0.93)**	0.77 (0.50–1.20)	0.83 (0.46–1.49)	0.87 (0.42–1.76)
NIVO vs. DAB+TRAM	1.06 (0.68–1.63)	0.65 (0.37–1.14)	0.51 (0.24–1.07)	0.44 (0.17–1.08)
NIVO vs. IPI (10 mg/kg)	0.80 (0.61–1.06)	0.88 (0.60–1.28)	0.93 (0.55–1.56)	0.96 (0.50–1.84)
NIVO vs. IFN	0.73 (0.51–1.05)	0.80 (0.51–1.27)	0.84 (0.47–1.53)	0.86 (0.42–1.78)

^a^The value in each cell represents the hazard ratio (95% credible interval) for the comparison of the treatments; bolded values are statistically significant at the 0.05 significance level

*DAB* dabrafenib, *DMFS* distant metastasis-free survival, *HR* hazard ratio, *IFN* interferon, *IPI* ipilimumab, *NIVO* nivolumab, *NMA* network meta-analysis, *OBS* observation, *PBO* placebo, *TRAM* trametinib

### Safety

Based on odds ratios estimated in the safety analyses, nivolumab was associated with lower rates of grade 3/4 AEs than the other active interventions (Additional file 4: Appendix D, Table D.1) and lower rates of discontinuations due to AEs than the other active interventions, with the exception of pembrolizumab (Additional file 4: Appendix D, Table D.2). Overall discontinuation rates with nivolumab were lower than those with ipilimumab 10 mg/kg or IFN and similar to those with the other treatment options (Additional file 4: Appendix D, Table D.3). Detailed results of the safety analyses are presented in Additional file 4: Appendix D.

## Discussion

This study assessed clinical outcomes with adjuvant nivolumab compared with other treatment options in patients with resected melanoma. In this NMA, nivolumab was associated with a reduction in the risk of recurrence or death compared with observation/placebo, ipilimumab, or historical treatments such as IFN or chemotherapy. The risk of recurrence was significantly lower with nivolumab than dabrafenib plus trametinib at 24 months and beyond. In addition, the risk of developing distant metastases was significantly lower with nivolumab than observation/placebo, ipilimumab, or IFN. A comparison between nivolumab and pembrolizumab did not show any differences in the risk of recurrence or distant metastases.

The limitations associated with the source of data and methods used in indirect treatment comparisons should be considered in interpreting these findings. Inherent to all SLRs is the risk of missing relevant articles due to publication after the search date, improper cataloguing within databases, and publication in non-academic literature. These limitations were mitigated by using a broad search strategy among three major databases and conducting individual searches of relevant conference proceedings.

The feasibility assessment that was conducted to explore heterogeneity in terms of trial, patient, treatment, and outcome characteristics identified some important differences between the treatments [16]. However, the current analysis assumed that the type of therapies in the other-chemotherapy group or the observation/placebo group did not act as a treatment-effect modifier. In addition, this study assessed the potential effect of differences in baseline disease stage through subgroup analysis, which showed that the results observed in patients with stage III/IV disease were consistent with the analyses that included all disease stages. A thorough exploration of *BRAF* mutation status was precluded by a lack of reporting in all but three studies.

Among the approved treatment options at the time of this analysis, pembrolizumab had the shortest follow-up duration. The pembrolizumab arm of KEYNOTE-054 had only 25 patients at risk at 27 months and 3 patients at risk at 30 months [28], whereas the nivolumab arm of CheckMate 238 had 249 patients at risk at 27 months and 243 patients at risk at 30 months [40]. The shorter duration of follow-up may have created biased estimates in the time-varying HRs.

Treatment effect was assessed using both constant and time-varying HRs to identify differences between the treatments. Time-dependent and constant HRs for RFS were similar in comparisons between nivolumab and ipilimumab and between nivolumab and pembrolizumab (immunotherapies), but comparisons were more complex when evaluating nivolumab versus dabrafenib plus trametinib (targeted therapy). Both the constant HR for RFS over the entire follow-up period and the time-varying HR for RFS at 12 months showed that the risk of recurrence with nivolumab was similar to that with dabrafenib plus trametinib. However, HRs at 24, 36, and 48 months indicated a higher reduction in the risk of recurrence with nivolumab than with dabrafenib plus trametinib as adjuvant treatment. On the basis of these analyses, compared with targeted therapies, the treatment effect with nivolumab appears to be durable, with a potential for lower risk of recurrence over the long term, which may be an important consideration in informing treatment choice.

The current study highlights the overall safety risks among the different adjuvant treatment options. The odds of experiencing grade 3/4 AEs were lower with nivolumab than with the other treatments. However, this analysis did not take into account the type of AEs or the time to resolution of specific AEs (eg, immune-mediated AEs associated with immunotherapies versus treatment-related AEs associated with targeted therapies).

Development of the current work was based on similar NMA studies that were published recently [18, 19]. As in our analysis, one study presented an SLR and an NMA that showed similar efficacy between dabrafenib plus trametinib and nivolumab using a constant HR over time [18]; however, this analysis did not include a time-varying HR, which was incorporated into the current study. In another NMA study, a time-varying HR analysis was conducted, but nivolumab was excluded as a comparator because of differences in the duration of ipilimumab treatment in the CheckMate 238 (1 year) and EORTC 18071 (3 years) trials [19], which were included in the current study. Although the majority of recent trials have administered adjuvant treatment for 1 year, treatment duration is unlikely to influence time-varying HRs. Moreover, the median number of ipilimumab doses was four in the EORTC 18071 study, and only a few patients received ipilimumab beyond 1 year.

## Conclusions

In summary, nivolumab is an adjuvant treatment option with a promising risk–benefit profile indicated for the treatment of patients with resected melanoma. This study provides comparative evidence for nivolumab versus other adjuvant treatment options, as well as placebo. The efficacy assessment reported here may support patient preferences and clinician choices for short-term versus long-term effectiveness. The current analysis is consistent with the established safety profile of nivolumab. Due to the potential occurrence of immune-mediated AEs, additional analyses may be warranted, such as long-term follow-up of safety data.

## Supplementary information

**Additional file 1: Appendix A.** Systematic literature review details and results. **Figure A.1.** Study selection flow diagram of updated SLR (May 2019). **Table A.1.** Study selection criteria to identify trials for the systematic literature review. **Table A.2.** Search strategy for EMBASE (EMBASE 1974 to October 11, 2017; Search executed: October 12, 2017). **Table A.3.** Search strategy for MEDLINE (Ovid MEDLINE(R) In-Process & Other Non-Indexed Citations, Ovid MEDLINE(R) Daily and Ovid MEDLINE(R) 1946 to Present; Search executed: October 12, 2017). **Table A.4.** Search strategy for Cochrane Register of Controlled Trials (Cochrane Register of Controlled Trials September 2017; Search executed: October 12, 2017). **Table A.5.** Search strategy for EMBASE (EMBASE 1974 to 2018 May 07; Search executed: May 8, 2018). **Table A.6.** Search strategy for MEDLINE (Ovid MEDLINE(R) In-Process & Other Non-Indexed Citations, Ovid MEDLINE(R) Daily and Ovid MEDLINE(R) 1946 to Present; Search executed: May 8, 2018). **Table A.7.** Search strategy for Cochrane Register of Controlled Trials (Cochrane Register of Controlled Trials March 2018; Search executed: May 8, 2018). **Table A.8.** Search strategy for EMBASE (EMBASE 1974 to 2019 April 30; Search executed: May 1, 2019). **Table A.9.** Search strategy for MEDLINE (Ovid MEDLINE(R) In-Process & Other Non-Indexed Citations, Ovid MEDLINE(R) Daily and Ovid MEDLINE(R) 1946 to Present; Search executed: May 1, 2019). **Table A.10.** Search strategy for Cochrane Register of Controlled Trials (Cochrane Register of Controlled Trials April 2019; Search executed: May 1, 2019). **Table A.11.** Patient characteristics in randomized controlled trials included in network meta-analyses. **Table A.12.** Cochrane risk of bias assessment of randomized controlled trials included in the feasibility assessment. **Table A.13.** Safety data from randomized controlled trials included in the feasibility assessment

**Additional file 2: Appendix B.** Consistency checks. **Table B.1.** Assessment of consistency; full analysis set, all studies

**Additional file 3: Appendix C.** Results of the stage III/IV subgroup analyses. **Table C.1.** Stage III/IV population – constant hazard ratios estimated from fixed-effects network meta-analysis for recurrence-free/disease-free survival. **Table C.2.** Stage III/IV population – constant hazard ratios estimated from fixed-effects network meta-analysis for distant metastasis-free survival

**Additional file 4: Appendix D.** Safety analyses. **Figure D.1.** Network diagrams of randomized controlled trials for subgroup. **Table D.1.** Stage II/III/IV population – odds ratios estimated from fixed-effects network meta-analysis for grade 3/4 adverse events analysis of safety outcomes. **Table D.2.** Stage II/III/IV population – odds ratios estimated from fixed-effects network meta-analysis for discontinuations due to adverse events. **Table D.3.** Stage II/III/IV population – odds ratios estimated from fixed-effects network meta-analysis for discontinuations

## Data Availability

All data generated or analyzed during this study are included in this published article and its supplementary information files. The Bristol Myers Squibb policy on data sharing may be found at https://www.bms.com/researchers-and-partners/independent-research/data-sharing-request-process.html.

## Abbreviations

AE: Adverse event
CrI: Credible interval
DFS: Disease-free survival
DMFS: Distant metastasis-free survival
FDA: Food and Drug Administration
HR: Hazard ratio
IFN: Interferon
KM: Kaplan–Meier
NMA: Network meta-analysis
PD: Programmed death
RCT: Randomized controlled trial
RFS: Recurrence-free survival
SLR: Systematic literature review
