# Supplementary material for: Comparative efficacy and safety of adjuvant nivolumab versus other treatments in adults with resected melanoma: a systematic literature review and network meta-analysis
Source: BMC Cancer. 2021 Jan 5;21:3. doi: 10.1186/s12885-020-07538-1 (PMC7784366; doi:10.1186/s12885-020-07538-1)
Supplement: Supplementary file 1 — Additional file 1: Appendix A. Systematic literature review details and results. Figure A.1. Study selection flow diagram of updated SLR (May 2019). Table A.1. Study selection criteria to identify trials for the systematic literature review. Table A.2. Search strategy for EMBASE (EMBASE 1974 to October 11, 2017; Search executed: October 12, 2017). Table A.3. Search strategy for MEDLINE (Ovid MEDLINE(R) In-Process & Other Non-Indexed Citations, Ovid MEDLINE(R) Daily and Ovid MEDLINE(R) 1946 to Present; Search executed: October 12, 2017). Table A.4. Search strategy for Cochrane Register of Controlled Trials (Cochrane Register of Controlled Trials September 2017; Search executed: October 12, 2017). Table A.5. Search strategy for EMBASE (EMBASE 1974 to 2018 May 07; Search executed: May 8, 2018). Table A.6. Search strategy for MEDLINE (Ovid MEDLINE(R) In-Process & Other Non-Indexed Citations, Ovid MEDLINE(R) Daily and Ovid MEDLINE(R) 1946 to Present; Search executed: May 8, 2018). Table A.7. Search strategy for Cochrane Register of Controlled Trials (Cochrane Register of Controlled Trials March 2018; Search executed: May 8, 2018). Table A.8. Search strategy for EMBASE (EMBASE 1974 to 2019 April 30; Search executed: May 1, 2019). Table A.9. Search strategy for MEDLINE (Ovid MEDLINE(R) In-Process & Other Non-Indexed Citations, Ovid MEDLINE(R) Daily and Ovid MEDLINE(R) 1946 to Present; Search executed: May 1, 2019). Table A.10. Search strategy for Cochrane Register of Controlled Trials (Cochrane Register of Controlled Trials April 2019; Search executed: May 1, 2019). Table A.11. Patient characteristics in randomized controlled trials included in network meta-analyses. Table A.12. Cochrane risk of bias assessment of randomized controlled trials included in the feasibility assessment. Table A.13. Safety data from randomized controlled trials included in the feasibility assessment [file 12885_2020_7538_MOESM1_ESM.pdf]

Appendix A: Systematic literature review details and results

Methods

The eligibility criteria for the SLR were defined in terms of the population, interventions, comparisons, outcomes, and study design (PICOS) structure outlined in **Table. A.1**.

Table A.1. A comprehensive systematic literature search of MEDLINE, EMBASE, and the Cochrane Central Register of Controlled Trials was conducted. The original searches were executed on October 12, 2017 with predefined search strategies and was updated on May 8, 2018 (see Table A.2 to Table A.7 for search strategy details). Additionally, manual searches of four conference proceedings were conducted: International Society for Quality of Life Research 2017, Society for Melanoma Research 2017, Society for Immunotherapy of Cancer 2017, and American Academy of Cancer Research 2015 to 2018.

Two investigators independently reviewed all abstracts and proceedings identified in the literature search. All studies identified as eligible during the abstract screening were screened at a full-text stage by the same two reviewers. At each stage, if any discrepancies occurred between the studies that the two investigators selected, a third investigator provided arbitration. The two investigators independently extracted data on patient and study characteristics including treatment regimen, method of administration, frequency of administration, duration of treatment, concomitant/background therapies, age, sex, race, disease stage, performance status, comorbidities, and prior treatment experience.

The following outcomes were extracted: overall survival (OS), recurrence-free survival or disease-free survival (RFS/DFS), distant metastasis-free survival (DMFS), global quality of life as measured by EORTC QLQ-C30, all-cause grade 3/4 adverse events (AEs), and discontinuations. Two reviewers independently extracted all data from the included studies and recorded it in a spreadsheet. A third reviewer checked all data extraction.

Table A.1

Study selection criteria to identify trials for the systematic literature review.

Criteria	Description
Population	Adults (aged 18 years and older) with<br>• Non-metastatic stage III melanoma[a]<br>• Non-metastatic stage IV melanoma
Interventions	Eligible interventions include adjuvant treatment (given after surgery) with one of the following[b]:<br>• Nivolumab<br>• Ipilimumab<br>• Pembrolizumab<br>• Dabrafenib in combination with trametinib<br>• All interferon alphas (including 2a, 2b, pegylated 2a or 2b, and high and low doses)
Comparators	Eligible comparators include the following:<br>• Any treatment listed as an eligible intervention<br>• Placebo<br>• Standard of care<br>• Watchful waiting
Outcomes	Studies must report at least one of the following outcomes at a time point of 12 months or later:<br>• Overall survival<br>• Recurrence-free survival or disease-free survival<br>• Distant metastasis-free survival<br>• Adverse events grade 3/4<br>• Overall discontinuations<br>• Discontinuations due to adverse events<br>• Global quality of life as measured by EORTC QLQ-C30
Study design	Only randomized controlled trials will be included
Language	Only studies published in English will be included

[a]Studies assessing combined stage II/III patients will be included in the SLR, validity of inclusion in analyses will be assessed in the feasibility assessment. [b]Patients with background therapy (such as chemotherapy) in combination with one of the interventions of interest are eligible. EORTC QLQ-C30: European Organisation for Research and Treatment of Cancer Core Quality of Life Questionnaire.

Table A.2

Search strategy for EMBASE (EMBASE 1974 to October 11, 2017; Search executed: October 12, 2017).

No	Criteria	Strings	Hits
1	Population	exp Skin Neoplasms/	206,816
2		exp melanoma/	133,929
3		((Skin adj Neoplasm$) or (Skin adj Cancer$) or (Skin adj Tumour$) or (Skin adj Carcinoma$) or (Skin adj Adenocarcinoma$) or (Skin adj Sarcoma$) or Melanoma).ti,ab.	147,990
4		or/1-3	344,369
5	Intervention	exp nivolumab/	5395
6		(nivolumab or Opdivo or MDX-1106 or MDX1106 or ONO-4538 or ONO4538 or BMS-936558 or BMS936558).ti,ab.	2753
7		exp ipilimumab/	7942
8		(ipilimumab or Yervoy or CTLA-4 or CTLA4 or MDX-010 or MDX010 or MDX-101 or MDX101).ti,ab.	13,640
9		exp pembrolizumab/	4300
10		(pembrolizumab or lambrolizumab or Keytruda or MK-3475 or MK3475).ti,ab.	2027
11		exp trametinib/	2716
12		(trametinib or Mekinist or JTP-74057 or JTP74057 or GSK-1120212 or GSK1120212).ti,ab.	1262
13		exp dabrafenib/	2538
14		(dabrafenib or Tafinlar or GSK-2118436 or GSK2118436).ti,ab.	1171
15		exp interferons/	463,668
16		(interferon alpha or interferon alpha or interferon$).ti,ab.	176,346
17		or/5-16	510355
18	Study design	Clinical trial/	957,375
19		Randomized controlled trial/	476,396
20		Controlled clinical trial/	450,873
21		Multicenter study/	167,819
22		Phase 3 clinical trial/	30,537
23		Phase 4 clinical trial/	2745
24		Exp randomization/	76,036
25		Single blind procedure/	29,845
26		Double blind procedure/	143,863
27		Crossover procedure	53,679

No	Criteria	Strings	Hits
28		Placebo/	315,178
29		Randomi?ed controlled trial$.tw.	169,116
30		Rct.tw.	25,996
31		(random$ adj2 allocat$).tw.	35,330
32		Single blind$.tw.	20,242
33		Double blind$.tw.	183,557
34		((treble or triple) adj blind$).tw.	748
35		Placebo$.tw.	263,368
36		Prospective study/	407,734
37		or/18-36	1,896,300
38		Case study/	50,411
39		Case report.tw.	351,006
40		Abstract report/ or letter/	1,036,903
41		Conference proceeding.pt.	0
42		Conference abstract.pt.	2,725,105
43		Editorial.pt.	549,558
44		Letter.pt.	994,139
45		Note.pt.	691,353
46		or/38-45	5,364,560
47		37 not 46	1,509,652
48	Combined criteria	4 and 17 and 47	4556
49		48 not (conference abstract or review or letter or meta-analysis or case report or case series or posters or News or Newspaper article or meeting abstracts or lectures or interview or historical article or handbooks or guidelines or guidebooks or essays or editorial or database or comment or clinical conference or catalogs or case reports or Short Survey or Note or Erratum or Chapter or book series or book or Conference Paper or Conference Proceeding: Article or Conference Proceeding: Conference Paper or Conference Proceeding: Editorial or Conference Proceeding: Note or Conference Proceeding: Review or Journal: Book or Journal: Conference Abstract or Journal: Conference Paper or Journal: Conference Review or Journal: Editorial or Journal: Erratum or Journal: Letter or Journal: Note or Journal: Patent or Journal: Press Release or Journal: Review or Journal: Short Survey or Report or Report: Article or Report: Editorial or Report: Letter or Report: Review or Trade Journal: Conference Paper or Trade Journal: Conference Review or Trade Journal: Editorial or Trade Journal: Erratum or Trade Journal: Note or Trade Journal: Letter or Trade Journal: Review or Trade Journal: Short Survey).pt.	2048
50	Limits	limit 49 to english language	1941

Table A.3

Search strategy for MEDLINE (Ovid MEDLINE(R) In-Process & Other Non-Indexed Citations, Ovid MEDLINE(R) Daily and Ovid MEDLINE(R) 1946 to Present; Search executed: October 12, 2017)

No.	Criteria	Strings	Hits
1	Population	exp Skin Neoplasms/	118,146
2		exp melanoma/	90,257
3		((Skin adj Neoplasm$) or (Skin adj Cancer$) or (Skin adj Tumour$) or (Skin adj Carcinoma$) or (Skin adj Adenocarcinoma$) or (Skin adj Sarcoma$) or Melanoma).ti,ab.	116,512
4		or/1-3	207,599
5	Intervention	(nivolumab or Opdivo or MDX-1106 or MDX1106 or ONO-4538 or ONO4538 or BMS-936558 or BMS936558).ti,ab.	1411
6		(ipilimumab or Yervoy or CTLA-4 or CTLA4 or MDX-010 or MDX010 or MDX-101 or MDX101).ti,ab.	8639
7		(pembrolizumab or lambrolizumab or Keytruda or MK-3475 or MK3475).ti,ab.	933
8		(dabrafenib or Tafinlar or GSK-2118436 or GSK2118436).ti,ab.	635
9		(trametinib or Mekinist or JTP-74057 or JTP74057 or GSK-1120212 or GSK1120212).ti,ab.	626
10		exp interferons/	132,389
11		(interferon alpha or interferon alpha or interferon$).ti,ab.	145,756
12		or/5-11	198,811
13	Study design	Randomized Controlled Trials as Topic/	121,739
14		randomized controlled trial/	496,708
15		random Allocation/	99,603
16		double Blind Method/	157,447
17		single Blind Method/	26,552
18		clinical trial/	547,944
19		clinical trial, phase i.pt.	19,999
20		clinical trial, phase ii.pt.	32,245
21		clinical trial, phase iii.pt.	15,264
22		clinical trial, phase iv.pt.	1626
23		controlled clinical trial.pt.	99,243
24		randomized controlled trial.pt.	496,708

No.	Criteria	Strings	Hits
25		multicenter study.pt.	248,785
26		clinical trial.pt.	547,944
27		exp Clinical Trials as topic/	332,032
28		or/13-27	1,314,469
29		(clinical adj trial$).tw.	321,055
30		((singl$ or doubl$ or treb$ or tripl$) adj (blind$3 or mask$3)).tw.	167,602
31		placebos/	36,429
32		placebo$.tw.	208,937
33		randomly allocated.tw.	25,043
34		(allocated adj2 random$).tw.	28,196
35		or/29-34	582,914
36		28 or 35	1,540,682
37		case report.tw.	275,193
38		letter/	1,022,789
39		historical article/	356,001
40		or/37-39	1,639,442
41		36 not 40	1,505,568
42	Combined criteria	4 and 12 and 41	2389
43	Limits	limit 42 to english language	2254

Table A.4

Search strategy for Cochrane Register of Controlled Trials (Cochrane Register of Controlled Trials September 2017; Search executed: October 12, 2017)

No.	Criteria	Strings	Hits
1	Population	exp Skin Neoplasms/	1211
2		exp melanoma/	1087
3		((Skin adj Neoplasm$) or (Skin adj Cancer$) or (Skin adj Tumour$) or (Skin adj Carcinoma$) or (Skin adj Adenocarcinoma$) or (Skin adj Sarcoma$) or Melanoma).ti,ab.	3436
4		or/1-3	3905
5	Intervention	(nivolumab or Opdivo or MDX-1106 or MDX1106 or ONO-4538 or ONO4538 or BMS-936558 or BMS936558).ti,ab.	386
6		(ipilimumab or Yervoy or CTLA-4 or CTLA4 or MDX-010 or MDX010 or MDX-101 or MDX101).ti,ab.	553
7		(pembrolizumab or lambrolizumab or Keytruda or MK-3475 or MK3475).ti,ab.	312
8		(trametinib or Mekinist or JTP-74057 or JTP74057 or GSK-1120212 or GSK1120212).ti,ab.	103
9		(dabrafenib or Tafinlar or GSK-2118436 or GSK2118436).ti,ab.	87
10		exp interferons/	4643
11		(interferon alpha or interferon alpha or interferon$).ti,ab.	10,354
12		or/5-11	12,187
13	Combined criteria	4 and 12	881
14	Limits	limit 13 to english language	698

Table A.5

Search strategy for EMBASE (EMBASE 1974 to 2018 May 07; Search executed: May 8, 2018)

No.	Criteria	Strings	Hits
1	Population	exp Skin Neoplasms/	170,534
2		exp melanoma/	141,517
3		((Skin adj Neoplasm$) or (Skin adj Cancer$) or (Skin adj Tumour$) or (Skin adj Carcinoma$) or (Skin adj Adenocarcinoma$) or (Skin adj Sarcoma$) or Melanoma).ti,ab.	153,314
4		or/1-3	314,659
5	Intervention	exp nivolumab/	7229
6		(nivolumab or Opdivo or MDX-1106 or MDX1106 or ONO-4538 or ONO4538 or BMS-936558 or BMS936558).ti,ab.	4009
7		exp ipilimumab/	8902
8		(ipilimumab or Yervoy or CTLA-4 or CTLA4 or MDX-010 or MDX010 or MDX-101 or MDX101).ti,ab.	15,195
9		exp pembrolizumab/	5792
10		(pembrolizumab or lambrolizumab or Keytruda or MK-3475 or MK3475).ti,ab.	2976
11		exp trametinib/	3104
12		(trametinib or Mekinist or JTP-74057 or JTP74057 or GSK-1120212 or GSK1120212).ti,ab.	1492
13		exp dabrafenib/	2824
14		(dabrafenib or Tafinlar or GSK-2118436 or GSK2118436).ti,ab.	1326
15		exp interferons/	476,645
16		(interferon alpha or interferon alpha or interferon$).ti,ab.	179,944
17		or/5-16	527,673
18	Study design	Clinical trial/	969,154
19		Randomized controlled trial/	501,444
20		Controlled clinical trial/	461,444
21		Multicenter study/	184,816
22		Phase 3 clinical trial/	33,982
23		Phase 4 clinical trial/	2955
24		Exp randomization/	78,193
25		Single blind procedure/	31,288
26		Double blind procedure/	149,577
27		Crossover procedure	55,410

No.	Criteria	Strings	Hits
28		Placebo/	324,796
29		Randomi?ed controlled trial$.tw.	180,665
30		Rct.tw.	28,337
31		(random$ adj2 allocat$).tw.	36,631
32		Single blind$.tw.	21,080
33		Double blind$.tw.	189,103
34		((treble or triple) adj blind$).tw.	795
35		Placebo$.tw.	273,736
36		Prospective study/	446,354
37		or/18-36	1,991,193
38		Case study/	54,243
39		Case report.tw.	362,697
40		Abstract report/ or letter/	1,059,268
41		Conference proceeding.pt.	0
42		Conference abstract.pt.	2,999,760
43		Editorial.pt.	565,196
44		Letter.pt.	1,019,617
45		Note.pt.	713,143
46		or/38-45	5,708,740
47		37 not 46	1,541,018
48	Combined criteria	4 and 17 and 47	4367
49		48 not (conference abstract or review or letter or meta-analysis or case report or case series or posters or News or Newspaper article or meeting abstracts or lectures or interview or historical article or handbooks or guidelines or guidebooks or essays or editorial or database or comment or clinical conference or catalogs or case reports or Short Survey or Note or Erratum or Chapter or book series or book or Conference Paper or Conference Proceeding: Article or Conference Proceeding: Conference Paper or Conference Proceeding: Editorial or Conference Proceeding: Note or Conference Proceeding: Review or Journal: Book or Journal: Conference Abstract or Journal: Conference Paper or Journal: Conference Review or Journal: Editorial or Journal: Erratum or Journal: Letter or Journal: Note or Journal: Patent or Journal: Press Release or Journal: Review or Journal: Short Survey or Report or Report: Article or Report: Editorial or Report: Letter or Report: Review or Trade Journal: Conference Paper or Trade Journal: Conference Review or Trade Journal: Editorial or Trade Journal: Erratum or Trade Journal: Note or Trade Journal: Letter or Trade Journal: Review or Trade Journal: Short Survey).pt.	1998

No.	Criteria	Strings	Hits
50	Limits	limit 49 to english language	1895
51		limit 50 to yr=2017-2018	173

Table A.6

Search strategy for MEDLINE (Ovid MEDLINE(R) In-Process & Other Non-Indexed Citations, Ovid MEDLINE(R) Daily and Ovid MEDLINE(R) 1946 to Present; Search executed: May 8, 2018)

No.	Criteria	Strings	Hits
1`	Population	exp Skin Neoplasms/	114,555
2		exp melanoma/	86,405
3		((Skin adj Neoplasm$) or (Skin adj Cancer$) or (Skin adj Tumour$) or (Skin adj Carcinoma$) or (Skin adj Adenocarcinoma$) or (Skin adj Sarcoma$) or Melanoma).ti,ab.	112,846
4		or/1-3	200,609
5	Intervention	(nivolumab or Opdivo or MDX-1106 or MDX1106 or ONO-4538 or ONO4538 or BMS-936558 or BMS936558).ti,ab.	1820
6		(ipilimumab or Yervoy or CTLA-4 or CTLA4 or MDX-010 or MDX010 or MDX-101 or MDX101).ti,ab.	8531
7		(pembrolizumab or lambrolizumab or Keytruda or MK-3475 or MK3475).ti,ab.	1211
8		(dabrafenib or Tafinlar or GSK-2118436 or GSK2118436).ti,ab.	652
9		(trametinib or Mekinist or JTP-74057 or JTP74057 or GSK-1120212 or GSK1120212).ti,ab.	636
10		exp interferons/	126,998
11		(interferon alpha or interferon alpha or interferon$).ti,ab.	140,570
12		or/5-11	192,522
13	Study design	Randomized Controlled Trials as Topic/	116,037
14		randomized controlled trial/	460,338
15		random Allocation/	94,117
16		double Blind Method/	145,676
17		single Blind Method/	25,111
18		clinical trial/	510,132
19		clinical trial, phase i.pt.	18,060
20		clinical trial, phase ii.pt.	29,170
21		clinical trial, phase iii.pt.	13,766
22		clinical trial, phase iv.pt.	1522
23		controlled clinical trial.pt.	92,385
24		randomized controlled trial.pt.	460,338

No.	Criteria	Strings	Hits
25		multicenter study.pt.	233,164
26		clinical trial.pt.	510,132
27		exp Clinical Trials as topic/	313,579
28		or/13-27	1,234,822
29		(clinical adj trial$).tw.	306,666
30		((singl$ or doubl$ or treb$ or tripl$) adj (blind$3 or mask$3)).tw.	156,492
31		placebos/	33,897
32		placebo$.tw.	194,987
33		randomly allocated.tw.	24,170
34		(allocated adj2 random$).tw.	27,202
35		or/29-34	552,511
36		28 or 35	1,456,341
37		case report.tw.	270,211
38		letter/	986,050
39		historical article/	344,724
40		or/37-39	1,586,814
41		36 not 40	1,423,126
42	Combined criteria	4 and 12 and 41	2207
43	Limits	limit 42 to english language	2073
44		limit 43 to yr=2017-2018	223

Table A.7

Search strategy for Cochrane Register of Controlled Trials (Cochrane Register of Controlled Trials March 2018; Search executed: May 8, 2018)

No.	Criteria	Strings	Hits
1	Population	exp Skin Neoplasms/	1250
2		exp melanoma/	1114
3		((Skin adj Neoplasm$) or (Skin adj Cancer$) or (Skin adj Tumour$) or (Skin adj Carcinoma$) or (Skin adj Adenocarcinoma$) or (Skin adj Sarcoma$) or Melanoma).ti,ab.	3652
4		or/1-3	4126
5	Intervention	(nivolumab or Opdivo or MDX-1106 or MDX1106 or ONO-4538 or ONO4538 or BMS-936558 or BMS936558).ti,ab.	526
6		(ipilimumab or Yervoy or CTLA-4 or CTLA4 or MDX-010 or MDX010 or MDX-101 or MDX101).ti,ab.	647
7		(pembrolizumab or lambrolizumab or Keytruda or MK-3475 or MK3475).ti,ab.	413
8		(trametinib or Mekinist or JTP-74057 or JTP74057 or GSK-1120212 or GSK1120212).ti,ab.	128
9		(dabrafenib or Tafinlar or GSK-2118436 or GSK2118436).ti,ab.	107
10		exp interferons/	4695
11		(interferon alpha or interferon alpha or interferon$).ti,ab.	10,554
12		or/5-11	12,677
13	Combined criteria	4 and 12	967
14	Limits	limit 13 to english language	841
15		limit 14 to yr=2017-2018	221

Table A.8

Search strategy for EMBASE (EMBASE 1974 to 2019 April 30; Search executed: May 1, 2019)

No.	Criteria	Strings	Hits
1	Population	exp Skin Neoplasms/	168,649
2	Population	exp melanoma/	145,320
3	Population	((Skin adj Neoplasm$) or (Skin adj Cancer$) or (Skin adj Tumour$) or (Skin adj Carcinoma$) or (Skin adj Adenocarcinoma$) or (Skin adj Sarcoma$) or Melanoma).ti,ab.	161,282
4	Combined criteria	or/1-3	318,535
5	Intervention	exp nivolumab/	11,079
6	Intervention	(nivolumab or Opdivo or MDX-1106 or MDX1106 or ONO-4538 or ONO4538 or BMS-936558 or BMS936558).ti,ab.	6420
7	Intervention	exp ipilimumab/	10,872
8	Intervention	(ipilimumab or Yervoy or CTLA-4 or CTLA4 or MDX-010 or MDX010 or MDX-101 or MDX101).ti,ab.	17,681
9	Intervention	exp pembrolizumab/	9111
10	Intervention	(pembrolizumab or lambrolizumab or Keytruda or MK-3475 or MK3475).ti,ab.	4886
11	Intervention	exp trametinib/	3848
12	Intervention	(trametinib or Mekinist or JTP-74057 or JTP74057 or GSK-1120212 or GSK1120212).ti,ab.	1882
13	Intervention	exp dabrafenib/	3348
14	Intervention	(dabrafenib or Tafinlar or GSK-2118436 or GSK2118436).ti,ab.	1591
15	Intervention	exp interferons/	503731
16	Intervention	(interferon alpha or interferon alpha or interferon$).ti,ab.	185611
17	Combined criteria	or/5-16	563,635
18	Study design	Clinical trial/	956,736
19	Study design	Randomized controlled trial/	545,818
20	Study design	Controlled clinical trial/	461,840
21	Study design	Multicenter study/	213,864
22	Study design	Phase 3 clinical trial/	39,457
23	Study design	Phase 4 clinical trial/	3377
24	Study design	Exp randomization/	82,297
25	Study design	Single blind procedure/	34,807
26	Study design	Double blind procedure/	159,700
27	Study design	Crossover procedure	58,880
28	Study design	Placebo/	332,889

29	Study design	Randomi?ed controlled trial$.tw.	200,270
30	Study design	Rct.tw.	32,052
31	Study design	(random$ adj2 allocat$).tw.	39,493
32	Study design	Single blind$.tw.	22,741
33	Study design	Double blind$.tw.	196,891
34	Study design	((treble or triple) adj blind$).tw.	949
35	Study design	Placebo$.tw.	287,920
36	Study design	Prospective study/	514,568
37	Combined criteria	or/18-36	2,121,103
38	Study design	Case study/	60,785
39	Study design	Case report.tw.	379,672
40	Study design	Abstract report/ or letter/	1,095,361
41	Study design	Conference proceeding.pt.	0
42	Study design	Conference abstract.pt.	3,390,909
43	Study design	Editorial.pt.	598,106
44	Study design	Letter.pt.	1,059,364
45	Study design	Note.pt.	749,085
46	Combined criteria	or/38-45	6,217,068
47	Combined criteria	37 not 46	1,602,410
48	Combined criteria	4 and 17 and 47	4498
49	Combined criteria	48 not (conference abstract or review or letter or meta-analysis or case report or case series or posters or News or Newspaper article or meeting abstracts or lectures or interview or historical article or handbooks or guidelines or guidebooks or essays or editorial or database or comment or clinical conference or catalogs or case reports or Short Survey or Note or Erratum or Chapter or book series or book or Conference Paper or Conference Proceeding: Article or Conference Proceeding: Conference Paper or Conference Proceeding: Editorial or Conference Proceeding: Note or Conference Proceeding: Review or Journal: Book or Journal: Conference Abstract or Journal: Conference Paper or Journal: Conference Review or Journal: Editorial or Journal: Erratum or Journal: Letter or Journal: Note or Journal: Patent or Journal: Press Release or Journal: Review or Journal: Short Survey or Report or Report: Article or Report: Editorial or Report: Letter or Report: Review or Trade Journal: Conference Paper or Trade Journal: Conference Review or Trade Journal: Editorial or Trade Journal: Erratum or Trade Journal: Note or Trade Journal: Letter or Trade Journal: Review or Trade Journal: Short Survey).pt.	1980

Table A.9

Search strategy for MEDLINE (Ovid MEDLINE(R) In-Process & Other Non-Indexed Citations, Ovid MEDLINE(R) Daily and Ovid MEDLINE(R) 1946 to Present; Search executed: May 1, 2019)

No.	Criteria	Strings	Hits
1	Population	exp Skin Neoplasms/	119,018
2	Population	exp melanoma/	89,978
3	Population	((Skin adj Neoplasm$) or (Skin adj Cancer$) or (Skin adj Tumour$) or (Skin adj Carcinoma$) or (Skin adj Adenocarcinoma$) or (Skin adj Sarcoma$) or Melanoma).ti,ab.	118,685
4	Combined criteria	or/1-3	209,221
5	Intervention	(nivolumab or Opdivo or MDX-1106 or MDX1106 or ONO-4538 or ONO4538 or BMS-936558 or BMS936558).ti,ab.	2710
6	Intervention	(ipilimumab or Yervoy or CTLA-4 or CTLA4 or MDX-010 or MDX010 or MDX-101 or MDX101).ti,ab.	9579
7	Intervention	(pembrolizumab or lambrolizumab or Keytruda or MK-3475 or MK3475).ti,ab.	1900
8	Intervention	(dabrafenib or Tafinlar or GSK-2118436 or GSK2118436).ti,ab.	744
9	Intervention	(trametinib or Mekinist or JTP-74057 or JTP74057 or GSK-1120212 or GSK1120212).ti,ab.	785
10	Intervention	exp interferons/	130,019
11	Intervention	(interferon alpha or interferon alpha or interferon$).ti,ab.	145,442
12	Combined criteria	or/5-11	200,668
13	Study design	Randomized Controlled Trials as Topic/	123,342
14	Study design	randomized controlled trial/	481,119
15	Study design	random Allocation/	98,800
16	Study design	double Blind Method/	151,012
17	Study design	single Blind Method/	26,667
18	Study design	clinical trial/	515,964
19	Study design	clinical trial, phase i.pt.	18,863
20	Study design	clinical trial, phase ii.pt.	30,502
21	Study design	clinical trial, phase iii.pt.	14,982
22	Study design	clinical trial, phase iv.pt.	1701
23	Study design	controlled clinical trial.pt.	93,049
24	Study design	randomized controlled trial.pt.	481,119
25	Study design	multicenter study.pt.	249,453
26	Study design	clinical trial.pt.	515,964

27	Study design	exp Clinical Trials as topic/	325,170
28	Combined criteria	or/13-27	1,290,556
29	Study design	(clinical adj trial$).tw.	331,419
30	Study design	((singl$ or doubl$ or treb$ or tripl$) adj (blind$3 or mask$3)).tw.	163,286
31	Study design	placebos/	34,328
32	Study design	placebo$.tw.	203,752
33	Study design	randomly allocated.tw.	26,123
34	Study design	(allocated adj2 random$).tw.	29,266
35	Combined criteria	or/29-34	587,205
36	Combined criteria	28 or 35	1,531,398
37	Study design	case report.tw.	275,418
38	Study design	letter/	1,025,227
39	Study design	historical article/	351,205
40	Combined criteria	or/37-39	1,636,972
41	Combined criteria	36 not 40	1,496,835
42	Combined criteria	4 and 12 and 41	2356
43	Limits	limit 42 to english language	2218

Table A.10

Search strategy for Cochrane Register of Controlled Trials (Cochrane Register of Controlled Trials April 2019; Search executed: May 1, 2019)

No.	Criteria	Strings	Hits
1	Population	exp Skin Neoplasms/	1410
2	Population	exp melanoma/	1561
3	Population	((Skin adj Neoplasm$) or (Skin adj Cancer$) or (Skin adj Tumour$) or (Skin adj Carcinoma$) or (Skin adj Adenocarcinoma$) or (Skin adj Sarcoma$) or Melanoma).ti,ab.	5511
4	Combined criteria	or/1-3	6056
5	Intervention	(pembrolizumab or lambrolizumab or Keytruda or MK-3475 or MK3475).ti,ab.	933
6	Intervention	(nivolumab or Opdivo or MDX-1106 or MDX1106 or ONO-4538 or ONO4538 or BMS-936558 or BMS936558).ti,ab.	1186
7	Intervention	(ipilimumab or Yervoy or CTLA-4 or CTLA4 or MDX-010 or MDX010 or MDX-101 or MDX101).ti,ab.	1186
8	Intervention	(trametinib or Mekinist or JTP-74057 or JTP74057 or GSK-1120212 or GSK1120212).ti,ab.	215
9	Intervention	(dabrafenib or Tafinlar or GSK-2118436 or GSK2118436).ti,ab.	174
10	Intervention	exp interferons/	5358
11	Intervention	(interferon alpha or interferon alpha or interferon$).ti,ab.	13,252
12	Combined criteria	or/5-11	16,956
13	Combined criteria	4 and 12	1457
14	Limits	limit 13 to english language	1073

Patient characteristics, risk of bias, and safety data

Table A.11

Patient characteristics in randomized controlled trials included in network meta-analyses

Trial ID	Intervention	N	Median age, years (range)	Male, n (%)	Race, n (%)		
					Caucasian	Asian	Hispanic
Trials included in final network meta-analyses							
ECOG 1697[1]	IFN alfa-2b (high-dose)	581	52 (10–85)	336 (58)	547 (94)	0	4 (1)
	Observation	569	52 (19–81)	320 (56)	536 (94)	2 (< 1)	4 (1)
Scottish Study[2]	IFN alfa-2b (low-dose)	47	NR	NR	NR	NR	NR
	Observation	49	NR	NR	NR	NR	NR
WHO Melanoma Programme Trial 16[3]	IFN alfa-2b (low-dose)	225	NR	131 (58)	NR	NR	NR
	Observation	219	NR	114 (52)	NR	NR	NR
EORTC 18071[4]	Ipilimumab	475	51 (20–84)[a]	296 (62)	470 (99)	1 (< 1)	NR
	Placebo	476	52 (18–78)[a]	293 (62)	476 (100)	0	NR
EORTC 18952[5]	IFN alfa-2b (high-dose/13 months)	553	49 (17–74)	312 (56)	NR	NR	NR
	IFN alfa-2b (low-dose/25 months)	556	50 (16–75)	308 (55)	NR	NR	NR
	Observation	279	47 (20–75)	152 (54)	NR	NR	NR
EORTC 18991[6]	PEG IFN alfa-2b	627	50 (19–70)	366 (58)	NR	NR	NR
	Observation	629	50 (18–70)	367 (58)	NR	NR	NR

Trial ID	Intervention	N	Median age, years (range)	Male, n (%)	Race, n (%)		
					Caucasian	Asian	Hispanic
KEYNOTE-054[7,8]	Pembrolizumab	514	54 (19–83)	324 (63)	NR	NR	NR
	Placebo	505	54 (19–83)	304 (60)	NR	NR	NR
S0008[9]	IFN alfa-2b (high-dose)	203	48 (12–73)	141 (69)	195 (96)	NR	NR
	Other chemotherapy	199	46 (10–74)	141 (71)	190 (95)	NR	NR
Garbe et al. (2008; DeCOG)[10]	IFN alfa-2a (low-dose)	146	NR	92 (63)	NR	NR	NR
	IFN alfa-2a + dacarbazine	148	NR	93 (63)	NR	NR	NR
	Observation	147	NR	85 (58)	NR	NR	NR
COMBI-AD[11]	Dabrafenib + trametinib	435	50 (18–89)	244 (56)	NR	NR	NR
	Placebo	432	51 (20–85)	239 (55)	NR	NR	NR
AIM HIGH Study[12]	IFN alfa-2a (low-dose)	338	51 (16–79)[a]	191 (56)	NR	NR	NR
	Observation	336	52 (15–85)[a]	190 (57)	NR	NR	NR
Nordic IFN Trial[13]	IFN alfa-2b (1 year)	285	53 (18–73)	177 (62)	–	–	–
	IFN alfa-2b (2 years)	286	51 (22–77)	183 (64)	–	–	–
	Observation	284	51 (18–76)	167 (59)	–	–	–
Kim et al. (2009)[14]	Cisplatin, vinblastine, dacarbazine, IFN alfa-2b, interleukin-2	71	NR	44 (62)	NR	NR	NR
	IFN alfa-2b (high-dose)	33	NR	17 (52)	NR	NR	NR

Trial ID	Intervention	N	Median age, years (range)	Male, n (%)	Race, n (%)		
					Caucasian	Asian	Hispanic
	IFN alfa-2b (low-dose)	33	NR	19 (58)	NR	NR	NR
ECOG 1690[15]	IFN alfa-2b (low-dose)	215	NR	147 (68)	NR	NR	NR
	IFN alfa-2b (high-dose)	215	NR	128 (60)	NR	NR	NR
	Observation	212	NR	140 (66)	NR	NR	NR
ECOG 1684[16]	IFN alfa-2b (high-dose)	143	NR	90 (63)	NR	NR	NR
	Observation	137	NR	79 (58)	NR	NR	NR
EORTC 18871[17]	rIFN alfa-2b	240	52 (14–84)	127 (53)	NR	NR	NR
	Observation	244	52 (14–84)	138 (57)	NR	NR	NR
Stadler et al. (2006)[18]	Dacarbazine + natural human IFN alfa	128	53 (25–86)[a]	73 (57)	NR	NR	NR
	Observation	124	54 (23–82)[a]	69 (56)	NR	NR	NR
E1609[19]	Ipilimumab (high-dose)	406	54 (19–80)	NR (63)	NR	NR	NR
	Ipilimumab (low-dose)	367	55 (19–80)	NR (67)	NR	NR	NR
CheckMate 238[20,21]	Nivolumab	452	56 (19–83)	258 (57)	NR	NR	NR
	Ipilimumab	453	54 (18–86)	269 (59)	NR	NR	NR
Trials excluded from final network meta-analyses							
Eigentler (2016; DeCOG)[22]	PEG IFN alfa-2a	451	NR	242 (54)	NR	NR	NR
	IFN alfa-2a (low-dose)	458	NR	234 (51)	NR	NR	NR
EADO study[23]	PEG IFN alfa-2b	443	53 (18–75)	266 (60)	NR	NR	NR

Trial ID	Intervention	N	Median age, years (range)	Male, n (%)	Race, n (%)		
					Caucasian	Asian	Hispanic
	IFN alfa-2b (low-dose)	453	55 (20–76)	236 (52)	NR	NR	NR
Lian et al. 2013[24]	IFN alfa-2b (high-dose)	63	55 (26–84)	25 (40)	NR	NR	NR
	Chemotherapy	63	59 (18–75)	23 (36)	NR	NR	NR
	Observation	63	57 (25–80)	28 (44)	NR	NR	NR
Sunbelt Melanoma Trial[25] (SLN positive)	IFN alfa-2b (high-dose)	112	46 (40–57)[b]	60 (54)	NR	NR	NR
	Observation	106	49 (39–56)[b]	60 (57)	NR	NR	NR
Sunbelt Melanoma Trial[25] (SLN negative)	IFN alfa-2b (high-dose)	184	49 (38–57)[b]	84 (46)	NR	NR	NR
	Observation	192	50 (42–59)[b]	106 (55)	NR	NR	NR
MM-ADJ-5[26]	IFN alfa-2b (high-dose)	316	50 (20–76)	186 (59)	94	NR	NR
	IFN alfa-2b (intermittent high-dose)	311	52 (19–76)	174 (56)	94	NR	NR
Tobin et al. (2018)[27]	Ipilimumab	6	50 (34–67)	3 (50)	NR	NR	NR
	Ipilimumab + ATRA	4	55 (48–60)	1 (25)	NR	NR	NR
Wang et al. (2015)[28]	IFN alfa-2b (high-dose)	96	49	51 (53)	NR	96 (100)	NR
	Observation	52	50	29 (56)	NR	52 (100)	NR

[a]Mean age.

[b]Interquartile range.

AJCC: American Joint Committee on Cancer; ATRA: all-trans retinoic acid; IFN: interferon; NR: not reported; PEG: pegylated.

Table A.12

Cochrane risk of bias assessment of randomized controlled trials included in the feasibility assessment

Trial ID	Interventions	Sequence generation	Allocation concealment	Blinding of participants and personnel	Blinding of outcome assessment	Incomplete outcome data	Selective outcome reporting	Other sources of bias
Trials included in final network meta-analyses								
ECOG 1697[1]	IFN alfa-2b (high-dose) vs. Observation	Unclear risk	Low risk	Low risk	Unclear risk	Unclear risk	Low risk	Low risk
Scottish Study[2]	IFN alfa-2b (low-dose) vs. Observation	Unclear risk	Low risk	Low risk	Unclear risk	Unclear risk	Unclear risk	High risk
WHO Melanoma Programme Trial 16[3]	IFN alfa-2b (Low-dose) vs. Observation	Low risk	Low risk	Low risk	Unclear risk	Low risk	Low risk	Low risk
EORTC 18071[4]	Ipilimumab vs. Placebo	Low risk	Low risk	Low risk	Low risk	Low risk	Unclear risk	Low risk
EORTC 18952[5]	IFN alfa-2b (High-dose/13months) vs. IFN alfa-2b (Low-dose/25months) vs. Observation	Low risk	Low risk	Low risk	Unclear risk	Low risk	Low risk	Unclear risk
EORTC 18991[6]	PEG IFN alfa-2b vs. Observation	Low risk	Low risk	Low risk	Unclear risk	Unclear risk	Low risk	Low risk
KEYNOTE-054[7,8]	Pembrolizumab vs Placebo	Low risk	Low risk	Low risk	Low risk	Low risk	Low risk	Low risk
S0008[9]	IFN alfa-2b (High-Dose) vs. Other chemotherapy	Low risk	Low risk	Low risk	Unclear risk	Low risk	Unclear risk	Low risk
Garbe 2008 (DeCOG)[10]	IFN alfa-2a (low-Dose) vs. IFN alfa-2a	Low risk	Low risk	Low risk	Unclear risk	Low risk	Low risk	Low risk

Trial ID	Interventions	Sequence generation	Allocation concealment	Blinding of participants and personnel	Blinding of outcome assessment	Incomplete outcome data	Selective outcome reporting	Other sources of bias
	+ dacarbazine vs. Observation							
AIM HIGH Study[12]	IFN alfa-2a (low-dose) vs. Observation	Unclear risk	Low risk	Low risk	Unclear risk	High risk	Low risk	Low risk
Nordic IFN Trial[13]	IFN alfa-2b (1year) vs. Observation	Low risk	Low risk	Low risk	High risk	Low risk	Low risk	Unclear risk
Kim et al. 2009[14]	Cisplatin, Vinblastine, Dacarbazine, IFN alfa-2b, Interleukin-2 vs. IFN alfa-2b (High-dose) vs. IFN alfa-2b (Low-dose)	Unclear risk	Low risk	Low risk	Unclear risk	Low risk	Low risk	Unclear risk
ECOG 1690[15]	IFN alfa-2b (Low-dose) vs. IFN alfa-2b (High-dose) vs. Observation	Low risk	Low risk	Low risk	Unclear risk	Low risk	Low risk	Low risk
ECOG 1684[16]	IFN alfa-2b (High-dose) vs. Observation	Low risk	Low risk	Low risk	Unclear risk	Low risk	Low risk	Low risk
EORTC 18871[17]	rIFN alfa-2b vs. Observation	Unclear risk	Low risk	Low risk	Unclear risk	Low risk	Low risk	Unclear risk
COMBI-AD[11,29]	Dabrafenib +Trametinib vs. Placebo	Unclear risk	Low risk	Low risk	Low risk	Low risk	Low risk	Low risk
Stadler et al. 2006[18]	Dacarbazine + natural human IFN alfa vs. Observation	Low risk	Low risk	Low risk	Unclear risk	Low risk	Unclear risk	Unclear risk
E1609[19]	Ipilimumab (High-Dose) vs. Ipilimumab	Unclear risk	Low risk	Low risk	High risk	Unclear risk	Low risk	Low risk

Trial ID	Interventions	Sequence generation	Allocation concealment	Blinding of participants and personnel	Blinding of outcome assessment	Incomplete outcome data	Selective outcome reporting	Other sources of bias
	(Low-Dose) vs. IFN alfa-2b (High-dose)							
CheckMate 238[20,21]	Nivolumab vs. Ipilimumab	Unclear risk	Low risk	Low risk	Low risk	Low risk	Low risk	Low risk
Trials excluded from final network meta-analyses								
Eigentler 2016 (DeCOG)[22]	PEG IFN alfa-2a vs. IFN alfa-2a (low-dose)	Unclear risk	Low risk	Low risk	Unclear risk	Unclear risk	Low risk	Unclear risk
EADO study[23]	PEG IFN alfa-2b vs. IFN alfa-2b (low-dose)	Low risk	Low risk	Low risk	Unclear risk	Low risk	Low risk	Unclear risk
Lian et al. 2013[24]	IFN alfa-2b (high-dose) vs. Chemotherapy vs. Observation vs.	Low risk	Low risk	Low risk	Unclear risk	Low risk	Low risk	Low risk
Sunbelt Melanoma Trial[25]	IFN alfa-2b (high-dose) vs. Observation	Low risk	Low risk	Low risk	Unclear risk	Low risk	Low risk	High risk
MM-ADJ-5[26]	IFN alfa-2b (high-dose) vs. IFN alfa-2b (Intermittent High-Dose)	Low risk	Low risk	Low risk	Unclear risk	Unclear risk	Low risk	Low risk
Tobin et al. 2018[27]	Ipilimumab vs Ipilimumab and ATRA	Low risk	Unclear risk	Low risk	Unclear risk	Unclear risk	Unclear risk	Unclear risk
Wang et al. 2015[28]	IFN alfa-2b (high-dose) vs. Observation	Unclear risk	Low risk	Low risk	Unclear risk	Low risk	Low risk	Low risk

BSC: best supportive care

Table A.13

Safety data from randomized controlled trials included in the feasibility assessment

Trial ID	Intervention	Grade 3/4 adverse events, n (%)	Discontinuations, n (%)	Discontinuations due to adverse events, n (%)
Trials included in final network meta-analyses				
ECOG 1697[1]	IFN alfa-2b (High-dose)	(57.9)	NR	38 (6.5)
	Observation	(4.6)	NR	0 (0)
Scottish study[2]	IFN alfa-2b (Low-dose)	NR	NR	NR
	Observation	NR	NR	NR
WHO Melanoma Programme Trial 16[3]	IFN alfa-2b (Low-dose)	0 (0)	0 (0)	0 (0)
	Observation	0 (0)	NR	0 (0)
EORTC 18071[4]	Ipilimumab	255 (54.1)	447	244
	Placebo	124 (26.2)	411	20
EORTC 18952[5]	IFN alfa-2b (High-dose/13months)	NR	534	61
	IFN alfa-2b (Low-dose/25months)	NR	535	70
	Observation	NR	250	0
EORTC 18991[6]	PEG IFN alfa-2b	397	NR	228
	Observation	100	NR	0
KEYNOTE-054[7,8]	Pembrolizumab	161 (31.6)	208	70 (13.8)
	Placebo	93 (18.5)	202	11 (2.2)
S0008[9]	IFN alfa-2b (High-Dose)	(76)	105	39
	Other chemotherapy	(64)	33	29
Garbe 2008 (DeCOG)[10]	IFN alfa-2a (Low-Dose)	13	NR	20
	IFN alfa-2a + dacarbazine	25	NR	16
	Observation	NR	NR	2

Trial ID	Intervention	Grade 3/4 adverse events, n (%)	Discontinuations, n (%)	Discontinuations due to adverse events, n (%)
AIM HIGH Study[12]	IFN alfa-2a (low-dose)	56	NR	50 (14.8)
	Observation	14	NR	NR
Nordic IFN Trial[13]	IFN alfa-2b (1year)	NR	NR	72
	Observation	NR	NR	0
Kim et al. 2009[14]	Cisplatin, Vinblastine, Dacarbazine, IFN alfa-2b, Interleukin-2	NR	NR	4 (6)
	IFN alfa-2b (high-dose)	NR	NR	4
	IFN alfa-2b (low-dose)	NR	NR	5
ECOG 1690[15]	IFN alfa-2b (low-dose)	NR	NR	NR
	IFN alfa-2b (high-dose)	NR	NR	NR
	Observation	NR	NR	NR
ECOG 1684[16]	IFN alfa-2b (High-dose)	(67)	NR	NR
	Observation	NR	NR	NR
EORTC 18871[17]	rIFN alfa-2b	NR	NR	11
	Observation	NR	NR	0
COMBI-AD[11,29]	Dabrafenib +Trametinib	180 (41)	NR	114
	Placebo	61 (14)	NR	12 (3)
Stadler et al. 2006[18]	Dacarbazine + natural human IFN alfa	NR	NR	7
	Observation	NR	NR	0
E1609[19]	Ipilimumab (High-Dose)	(65.4)	NR	(53.7)
	Ipilimumab (Low-Dose)	(53.3)	NR	(34.9)
	IFN alfa-2b (High-dose)	NR	NR	NR
CheckMate 238[20,21]	Nivolumab	115 (25.4)	177	44
	Ipilimumab	250 (55.2)	331	193
Trials excluded from final network meta-analyses				

Trial ID	Intervention	Grade 3/4 adverse events, n (%)	Discontinuations, n (%)	Discontinuations due to adverse events, n (%)
Eigentler 2016 (DeCOG)[22]	PEG IFN alfa-2a	NR	260	118
	IFN alfa-2a (low-dose)	NR	235	61
EADO study[23]	PEG IFN alfa-2b	(47.3)	235	122
	IFN alfa-2b (low-dose)	(25.2)	136	58
Lian et al. 2013[24]	IFN alfa-2b (high-dose)	NR	1	1
	Chemotherapy	NR	NR	NR
	Observation	NR	2	2
Sunbelt Melanoma Trial[25]	IFN alfa-2b (high-dose)	NR	NR	NR
	Observation	NR	NR	NR
MM-ADJ-5[26]	IFN alfa-2b (high-dose)	NR	NR	46
	IFN alfa-2b (Intermittent high-dose)	NR	NR	21
Tobin et al. 2018[27]	Ipilimumab	2 (33)	NR	NR
	Ipilimumab and ATRA	3 (75)	NR	NR
Wang et al. 2015[28]	IFN alfa-2b (High-dose)	NR	NR	5
	Observation	NR	NR	NR

BSC: best supportive care; NR: not reported.

References

1. Agarwala SS, Lee SJ, Yip W, Rao UN, Tarhini AA, Cohen GI, et al. Phase III randomized study of 4 weeks of high-dose interferon-α-2b in stage T2bNO, T3a-bNO, T4a-bNO, and T1-4N1a-2a (microscopic) melanoma: a trial of the Eastern Cooperative Oncology Group–American College of Radiology Imaging Network Cancer Research Group (E1697). J Clin Oncol. 2017;35:885–92. doi: 10.1200/JCO.2016.70.2951.

2. Cameron DA, Cornbleet MC, Mackie RM, Hunter JA, Gore M, Hancock B, et al; Scottish Melanoma Group. Adjuvant interferon alpha 2b in high risk melanoma — the Scottish study. Br J Cancer. 2001;84:1146–9.

3. Cascinelli N, Belli F, MacKie RM, Santinami M, Bufalino R, Morabito A. Effect of long-term adjuvant therapy with interferon alpha-2a in patients with regional node metastases from cutaneous melanoma: a randomised trial. Lancet. 2001;358:866–9.

4. Eggermont AM, Chiarion-Sileni V, Grob JJ, Dummer R, Wolchok JD, Schmidt H, et al. Adjuvant ipilimumab versus placebo after complete resection of high-risk stage III melanoma (EORTC 18071): a randomised, double-blind, phase 3 trial. Lancet Oncol. 2015;16:522–30. doi: 10.1016/S1470-2045(15)70122-1.

5. Eggermont AM, Suciu S, MacKie R, Ruka W, Testori A, Kruit W, et al; EORTC Melanoma Group. Post-surgery adjuvant therapy with intermediate doses of interferon alfa 2b versus observation in patients with stage IIb/III melanoma (EORTC 18952): randomised controlled trial. Lancet. 2005;366:1189–96.

6. Eggermont AM, Suciu S, Santinami M, Testori A, Kruit WH, Marsden J, et al; EORTC Melanoma Group. Adjuvant therapy with pegylated interferon alfa-2b versus observation alone in resected stage III melanoma: final results of EORTC 18991, a randomised phase III trial. Lancet. 2008;372:117–26. doi: 10.1016/S0140-6736(08)61033-8.

7. European Medicines Agency. Keytruda: European Public Assessment Report (EPAR) - Product Information. 2019. https://www.ema.europa.eu/en/documents/product-information/keytruda-epar-product-information_en.pdf. Accessed 30 Sept 2019.

8. Eggermont AMM, Blank CU, Mandala M, Long GV, Atkinson V, Dalle S, et al. Adjuvant pembrolizumab versus placebo in resected stage III melanoma. N Engl J Med. 2018;378:1789–801.

9. Flaherty LE, Othus M, Atkins MB, Tuthill RJ, Thompson JA, Vetto JT, et al. Southwest Oncology Group S0008: a phase III trial of high-dose interferon alfa-2b versus cisplatin, vinblastine, and dacarbazine, plus interleukin-2 and interferon in patients with high-risk melanoma—an intergroup study of Cancer and Leukemia Group B, Children's Oncology Group, Eastern Cooperative Oncology Group, and Southwest Oncology Group. J Clin Oncol. 2014;32:3771–8. doi: 10.1200/JCO.2013.53.1590.

10. Garbe C, Radny P, Linse R, Dummer R, Gutzmer R, Ulrich J, et al. Adjuvant low-dose interferon α2a with or without dacarbazine compared with surgery alone: a prospective-randomized phase III DeCOG trial in melanoma patients with regional lymph node metastasis. Ann Oncol. 2008;19:1195–201. doi: 10.1093/annonc/mdn001.

11. Long GV, Hauschild A, Santinami M, Atkinson V, Mandalà M, Chiarion-Sileni V, et al. Adjuvant dabrafenib plus trametinib in stage III BRAF-mutated melanoma. N Engl J Med. 2017;377:1813–23. doi: 10.1056/NEJMoa1708539.

12. Hancock BW, Wheatley K, Harris S, Ives N, Harrison G, Horsman JM, et al. Adjuvant interferon in high-risk melanoma: the AIM HIGH Study—United Kingdom Coordinating Committee on Cancer Research randomized study of adjuvant low-dose extended-duration interferon alfa-2a in high-risk resected malignant melanoma. J Clin Oncol. 2004;22:53–61.

13. Hansson J, Aamdal S, Bastholt L, Brandberg Y, Hernberg M, Nilsson B, et al; Nordic Melanoma Cooperative Group. Two different durations of adjuvant therapy with intermediate-dose interferon alfa-2b in patients with high-risk melanoma (Nordic IFN trial): a randomised phase 3 trial. Lancet Oncol. 2011;12:144–52. doi: 10.1016/S1470-2045(10)70288-6.

14. Kim KB, Legha SS, Gonzalez R, Anderson CM, Johnson MM, Liu P, et al. A randomized phase III trial of biochemotherapy versus interferon-alpha- 2b for adjuvant therapy in patients at high risk for melanoma recurrence. Melanoma Res. 2009;19:42–9. doi: 10.1097/CMR.0b013e328314b84a.

15. Kirkwood JM, Ibrahim JG, Sondak VK, Richards J, Flaherty LE, Ernstoff MS, et al. High- and low-dose interferon alfa-2b in high-risk melanoma: first analysis of intergroup trial E1690/S9111/C9190. J Clin Oncol. 2000;18:2444–58.

16. Kirkwood JM, Strawderman MH, Ernstoff MS, Smith TJ, Borden EC, Blum RH. Interferon alfa-2b adjuvant therapy of high-risk resected cutaneous melanoma: the Eastern Cooperative Oncology Group Trial EST 1684. J Clin Oncol. 1996;14:7–17.

17. Kleeberg UR, Suciu S, Bröcker EB, Ruiter DJ, Chartier C, Lienard D, et al; EORTC Melanoma Group in cooperation with the German Cancer Society (DKG). Final results of the EORTC 18871/DKG 80-1 randomised phase III trial: rIFN-alpha2b versus rIFN-gamma versus ISCADOR M versus observation after surgery in melanoma patients with either high-risk primary (thickness >3 mm) or regional lymph node metastasis. Eur J Cancer. 2004;40:390–402.

18. Stadler R, Luger T, Bieber T, Köhler U, Linse R, Technau K, et al. Long-term survival benefit after adjuvant treatment of cutaneous melanoma with dacarbazine and low dose natural interferon alpha: a controlled, randomised multicentre trial. Acta Oncol. 2006;45:389–99.

19. Tarhini AA, Lee SJ, Hodi FS, Rao UNM, Cohen GI, Hamid O, et al. A phase III randomized study of adjuvant ipilimumab (3 or 10 mg/kg) versus high-dose interferon alfa-2b for resected high-risk melanoma (U.S. Intergroup E1609): preliminary safety and efficacy of the ipilimumab arms. J Clin Oncol. 2017;35(Suppl). Abstract 9500. doi: 10.1200/JCO.2017.35.15_suppl.9500.

20. Weber J, Mandala M, Del Vecchio M, Gogas HJ, Arance AM, Cowey CL, et al. Adjuvant nivolumab versus ipilimumab in resected stage III or IV melanoma. N Engl J Med. 2017;377:1824–35. doi: 10.1056/NEJMoa1709030.

21. Weber J, Del Vecchio M, Mandala M, Gogas HJ, Arance AM, Dalle S, et al. Adjuvant nivolumab versus ipilimumab in resected stage III/IV melanoma: 3-year efficacy and biomarker results from the phase 3 CheckMate 238 trial. Presented at ESMO 2019; 27 Sept–1 Oct 2019; Barcelona, Spain.

22. Eigentler TK, Gutzmer R, Hauschild A, Heinzerling L, Schadendorf D, Nashan D, et al; Dermatologic Cooperative Oncology Group (DeCOG). Adjuvant treatment with pegylated interferon α-2a versus low-dose interferon α-2a in patients with high-risk melanoma: a randomized phase III DeCOG trial. Ann Oncol. 2016;27:1625–32. doi: 10.1093/annonc/mdw225.

23. Grob JJ, Jouary T, Dréno B, Asselineau J, Gutzmer R, Hauschild A, et al. Adjuvant therapy with pegylated interferon alfa-2b (36 months) versus low-dose interferon alfa-2b (18 months) in melanoma patients without macrometastatic nodes: an open-label,

randomised, phase 3 European Association for Dermato-Oncology (EADO) study. Eur J Cancer. 2013;49:166–74. doi: 10.1016/j.ejca.2012.07.018.

24. Lian B, Si L, Cui C, Chi Z, Sheng X, Mao L, et al. Phase II randomized trial comparing high-dose IFN-α2b with temozolomide plus cisplatin as systemic adjuvant therapy for resected mucosal melanoma. Clin Cancer Res. 2013;19:4488–98. doi: 10.1158/1078-0432.CCR-13-0739.

25. McMasters KM, Egger ME, Edwards MJ, Ross MI, Reintgen DS, Noyes RD, et al. Final Results of the Sunbelt Melanoma Trial: a multi-institutional prospective randomized phase III study evaluating the role of adjuvant high-dose interferon alfa-2b and completion lymph node dissection for patients staged by sentinel lymph node biopsy. J Clin Oncol. 2016;34:1079–86. doi: 10.1200/JCO.2015.63.3776.

26. Mohr P, Hauschild A, Trefzer U, Enk A, Tilgen W, Loquai C, et al. Intermittent high-dose intravenous interferon alfa-2b for adjuvant treatment of stage III melanoma: final analysis of a randomized phase III Dermatologic Cooperative Oncology Group Trial. J Clin Oncol. 2015;33:4077–84. doi: 10.1200/JCO.2014.59.6932.

27. Tobin RP, Jordan KR, Robinson WA, Davis D, Borges VF, Gonzalez R, et al. Targeting myeloid-derived suppressor cells using all-trans retinoic acid in melanoma patients treated with Ipilimumab. Int Immunopharmacol. 2018;63:282–91. doi: 10.1016/j.intimp.2018.08.007.

28. Wang X, Mao LL, Si L, Chi Z, Cui C, Nan X, et al. Efficacy of high-dose adjuvant interferon therapy in high-risk melanoma harboring gene mutations. J Clin Oncol. 2015;33(Suppl). Abstract 9047. doi: 10.1200/jco.2015.33.15_suppl.9047.

29. Hauschild A, Dummer R, Schadendorf D, Santinami M, Atkinson V, Mandalà M, et al. Longer follow-up confirms relapse-free survival benefit with adjuvant dabrafenib plus trametinib in patients with resected *BRAF* V600-mutant stage III melanoma. J Clin Oncol. 2018;36:3441–9. doi: 10.1200/JCO.18.01219.
