# Supplementary material for: Comparative efficacy and safety of adjuvant nivolumab versus other treatments in adults with resected melanoma: a systematic literature review and network meta-analysis
Source: BMC Cancer. 2021 Jan 5;21:3. doi: 10.1186/s12885-020-07538-1 (PMC7784366; doi:10.1186/s12885-020-07538-1)
Supplement: Supplementary file 2 — Additional file 2: Appendix B. Consistency checks. Table B.1. Assessment of consistency; full analysis set, all studies [file 12885_2020_7538_MOESM2_ESM.pdf]

Appendix B: Consistency checks

For the primary analysis, one close loop existed (interferon (IFN) – other chemotherapy – observation or placebo). In order to assess consistency, the trials informing the direct comparison of other chemotherapy vs observation or placebo (Garbe 2008 [DeCOG][1] and Stadler 2006[2]) were removed. The resulting other chemotherapy vs observation or placebo hazard ratio (HR) was estimated using only indirect evidence. This value was then compared to the HR from the primary analysis that includes direct evidence. For both outcomes, the HRs in the two scenarios were in the same direction and there are no inconsistencies.

Table B.1

Assessment of consistency; full analysis set, all studies

Closed loop	Direct evidence trials removed	Comparison assessed	Direct evidence<br>HR (95% CrI)	Indirect evidence<br>HR (95% CrI)
IFN - Other chemotherapy - observation or placebo	Garbe 2008 (DeCOG)[1], Stadler 2006[2]	Other chemotherapy vs observation or placebo	OS 0.94 (0.79, 1.12)<br>RFS 0.85 (0.71, 1.03)	OS 0.92 (0.68, 1.23)<br>RFS 0.69 (0.54, 0.89)

CrI: credible interval; HR: hazard ratio; IFN: interferon; OS: overall survival; RFS: recurrence-free survival.

References

1. Garbe C, Radny P, Linse R, Dummer R, Gutzmer R, Ulrich J, et al. Adjuvant low-dose interferon α2a with or without dacarbazine compared with surgery alone: a prospective-randomized phase III DeCOG trial in melanoma patients with regional lymph node metastasis. Ann Oncol. 2008;19:1195–201. doi: 10.1093/annonc/mdn001.

2. Stadler R, Luger T, Bieber T, Köhler U, Linse R, Technau K, et al. Long-term survival benefit after adjuvant treatment of cutaneous melanoma with dacarbazine and low dose natural interferon alpha: a controlled, randomised multicentre trial. Acta Oncol. 2006;45:389–99.
