# Supplementary material for: Comparative efficacy and safety of adjuvant nivolumab versus other treatments in adults with resected melanoma: a systematic literature review and network meta-analysis
Source: BMC Cancer. 2021 Jan 5;21:3. doi: 10.1186/s12885-020-07538-1 (PMC7784366; doi:10.1186/s12885-020-07538-1)
Supplement: Supplementary file 3 — Additional file 3: Appendix C. Results of the stage III/IV subgroup analyses. Table C.1. Stage III/IV population – constant hazard ratios estimated from fixed-effects network meta-analysis for recurrence-free/disease-free survival. Table C.2. Stage III/IV population – constant hazard ratios estimated from fixed-effects network meta-analysis for distant metastasis-free survival [file 12885_2020_7538_MOESM3_ESM.pdf]

Appendix C: Results of the stage III/IV subgroup analyses

Table C.1

Constant hazard ratio estimates[a] from a fixed-effects network meta-analysis of recurrence-free/disease-free survival in patients with resected stage III/IV melanoma.

Observation or placebo	**1.94 (1.50, 2.49)**	**2.04 (1.68, 2.48)**	**1.79 (1.46, 2.19)**	**1.32 (1.11, 1.55)**	1.31 (1.00, 1.72)	1.15 (0.95, 1.40)	**1.11 (1.01, 1.22)**
**0.52 (0.40, 0.67)**	Nivolumab	1.06 (0.77, 1.46)	0.92 (0.67, 1.28)	**0.68 (0.56, 0.82)**	**0.68 (0.51, 0.90)**	**0.60 (0.43, 0.82)**	**0.57 (0.44, 0.75)**
**0.49 (0.40, 0.60)**	0.95 (0.69, 1.30)	Dabrafenib + trametinib	0.87 (0.66, 1.16)	**0.65 (0.50, 0.83)**	**0.64 (0.46, 0.90)**	**0.56 (0.43, 0.75)**	**0.54 (0.44, 0.68)**
**0.56 (0.46, 0.69)**	1.08 (0.78, 1.50)	1.15 (0.86, 1.51)	Pembrolizumab	**0.74 (0.57, 0.96)**	0.74 (0.53, 1.04)	**0.65 (0.49, 0.86)**	**0.62 (0.50, 0.78)**
**0.76 (0.65, 0.90)**	**1.47 (1.22, 1.78)**	**1.55 (1.21, 2.00)**	**1.36 (1.04, 1.76)**	Ipilimumab 10 mg/kg	1.00 (0.81, 1.24)	0.88 (0.67, 1.14)	0.84 (0.70, 1.03)
0.76 (0.58, 1.00)	**1.47 (1.11, 1.96)**	**1.55 (1.11, 2.17)**	1.36 (0.96, 1.90)	1.00 (0.81, 1.24)	Ipilimumab 3 mg/kg	0.88 (0.63, 1.24)	0.84 (0.63, 1.13)
0.87 (0.72, 1.05)	**1.68 (1.22, 2.31)**	**1.77 (1.34, 2.33)**	**1.55 (1.16, 2.06)**	1.14 (0.88, 1.48)	1.14 (0.81, 1.60)	Other chemotherapy	0.96 (0.80, 1.15)
**0.90 (0.82, 0.99)**	**1.74 (1.33, 2.28)**	**1.84 (1.48, 2.28)**	**1.61 (1.28, 2.02)**	1.19 (0.97, 1.44)	1.19 (0.88, 1.59)	1.04 (0.87, 1.25)	IFN pooled

[a]The value in each cell represents the hazard ratio (95% credible interval) for the comparison of the treatment indicated in that row versus the treatment indicated in that column; bolded values are statistically significant at the 0.05 significance level.

IFN: interferon.

Table C.2

Constant hazard ratio estimates[a] from a fixed-effects network meta-analysis of distant metastasis-free survival in patients with stage III/IV melanoma.

Observation or placebo	**1.69 (1.26, 2.24)**	**1.89 (1.49, 2.37)**	**1.32 (1.11, 1.56)**	1.06 (0.94, 1.20)
**0.59 (0.45, 0.79)**	Nivolumab	1.12 (0.77, 1.62)	**0.78 (0.62, 0.98)**	**0.63 (0.46, 0.86)**
**0.53 (0.42, 0.67)**	0.90 (0.62, 1.29)	Dabrafenib + trametinib	**0.70 (0.52, 0.94)**	**0.56 (0.43, 0.73)**
**0.76 (0.64, 0.90)**	**1.28 (1.02, 1.61)**	**1.43 (1.07, 1.91)**	Ipilimumab 10 mg/kg	0.81 (0.65, 1.00)
0.94 (0.83, 1.06)	**1.60 (1.16, 2.17)**	**1.78 (1.36, 2.31)**	1.24 (1.00, 1.53)	IFN pooled

[a]The value in each cell represents the hazard ratio (95% credible interval) for the comparison of the treatment indicated in that row versus the treatment indicated in that column; bolded values are statistically significant at the 0.05 significance level.

IFN: interferon.
