# Supplementary material for: Comparative efficacy and safety of adjuvant nivolumab versus other treatments in adults with resected melanoma: a systematic literature review and network meta-analysis
Source: BMC Cancer. 2021 Jan 5;21:3. doi: 10.1186/s12885-020-07538-1 (PMC7784366; doi:10.1186/s12885-020-07538-1)
Supplement: Supplementary file 4 — Additional file 4: Appendix D. Safety analyses. Figure D.1. Network diagrams of randomized controlled trials for subgroup. Table D.1. Stage II/III/IV population – odds ratios estimated from fixed-effects network meta-analysis for grade 3/4 adverse events analysis of safety outcomes. Table D.2. Stage II/III/IV population – odds ratios estimated from fixed-effects network meta-analysis for discontinuations due to adverse events. Table D.3. Stage II/III/IV population – odds ratios estimated from fixed-effects network meta-analysis for discontinuations [file 12885_2020_7538_MOESM4_ESM.pdf]

Appendix D: Safety analyses

Figure D.1

Network diagrams of randomized controlled trials for subgroup analysis of safety outcomes

A. Stage II/III/IV population – grade 3/4 adverse events

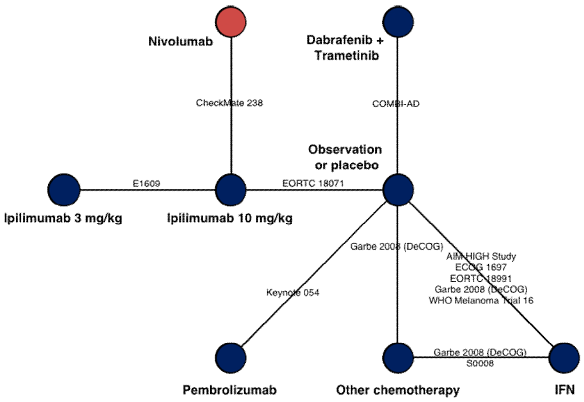

B. Stage II/III/IV population – discontinuations due to adverse events

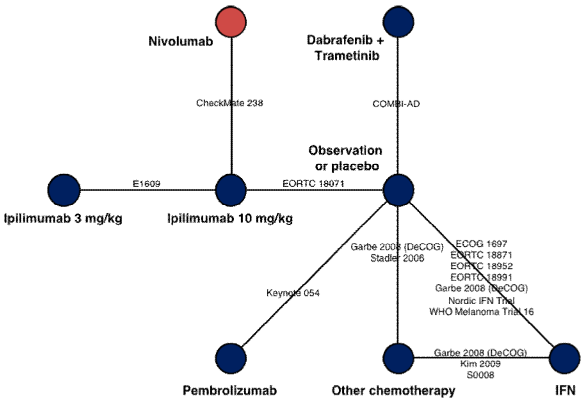

C. Stage III/IV subgroup population – discontinuations

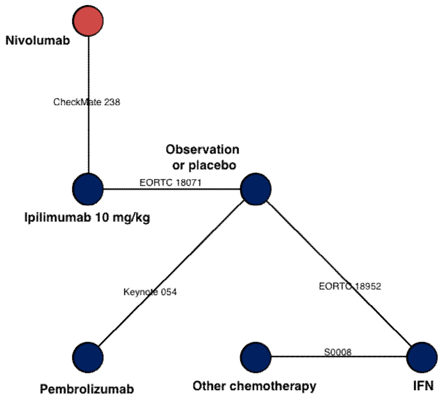

Notes: Trial names in red font indicate stage III subgroup data was used to inform comparison; other chemotherapy includes various combinations of cisplatin, vinblastine, dacarbazine, interleukin-2, temozolomide, and IFN-alfa-2b. IFN: interferon.

Table D.1

Odds ratio estimates[a] from a fixed-effects network meta-analysis of grade 3/4 adverse events in patients with stage II-IV melanoma.

Observation or placebo	1.08 (0.73, 1.60)	**0.23 (0.16, 0.32)**	**0.30 (0.23, 0.39)**	**0.50 (0.34, 0.72)**	**0.05 (0.03, 0.08)**	**0.09 (0.07, 0.11)**	**0.49 (0.37, 0.66)**
0.92 (0.63, 1.37)	Nivolumab	**0.21 (0.13, 0.36)**	**0.28 (0.21, 0.36)**	**0.46 (0.32, 0.66)**	**0.05 (0.03, 0.08)**	**0.08 (0.05, 0.13)**	**0.45 (0.28, 0.74)**
**4.32 (3.12, 6.06)**	**4.68 (2.79, 7.86)**	Dabrafenib + trametinib	1.30 (0.84, 1.99)	**2.15 (1.31, 3.54)**	**0.21 (0.12, 0.37)**	**0.40 (0.27, 0.59)**	**2.13 (1.37, 3.30)**
**3.34 (2.55, 4.41)**	**3.62 (2.74, 4.79)**	0.77 (0.50, 1.18)	Ipilimumab 10 mg/kg	**1.66 (1.29, 2.13)**	**0.16 (0.10, 0.27)**	**0.31 (0.22, 0.43)**	**1.64 (1.10, 2.46)**
**2.01 (1.40, 2.92)**	**2.18 (1.50, 3.17)**	**0.47 (0.28, 0.76)**	**0.60 (0.47, 0.77)**	Ipilimumab 3 mg/kg	**0.10 (0.06, 0.17)**	**0.18 (0.12, 0.28)**	0.99 (0.62, 1.59)
**20.46 (13.30, 31.55)**	**22.15 (12.44, 39.52)**	**4.73 (2.72, 8.13)**	**6.12 (3.69, 10.18)**	**10.16 (5.74, 17.93)**	Other chemotherapy	**1.88 (1.28, 2.75)**	**10.05 (5.97, 17.04)**
**10.91 (8.90, 13.40)**	**11.81 (7.61, 18.32)**	**2.52 (1.70, 3.69)**	**3.26 (2.33, 4.57)**	**5.41 (3.56, 8.23)**	**0.53 (0.36, 0.78)**	IFN	**5.36 (3.75, 7.66)**
**2.03 (1.52, 2.73)**	**2.20 (1.34, 3.59)**	**0.47 (0.30, 0.73)**	**0.61 (0.41, 0.91)**	1.01 (0.63, 1.62)	**0.10 (0.06, 0.17)**	**0.19 (0.13, 0.27)**	Pembrolizumab

[a]The value in each cell represents the hazard ratio (95% credible interval) for the comparison of the treatment indicated in that row versus the treatment indicated in that column; bolded values are statistically significant at the 0.05 significance level.

IFN: interferon.

Table D.2

Odds ratio estimates[a] from a fixed-effects network meta-analysis of discontinuations due to adverse events in patients with stage II-IV melanoma.

Observation or placebo	**0.28 (0.15, 0.51)**	**0.08 (0.04, 0.14)**	**0.04 (0.02, 0.07)**	**0.09 (0.05, 0.15)**	**0.07 (0.04, 0.10)**	**0.04 (0.03, 0.06)**	**0.14 (0.07, 0.25)**
**3.52 (1.95, 6.48)**	Nivolumab	**0.28 (0.11, 0.65)**	**0.14 (0.10, 0.21)**	**0.31 (0.20, 0.49)**	**0.23 (0.11, 0.49)**	**0.15 (0.08, 0.30)**	0.48 (0.19, 1.15)
**12.65 (7.16, 24.76)**	**3.62 (1.53, 8.82)**	Dabrafenib + trametinib	0.52 (0.24, 1.18)	1.12 (0.50, 2.67)	0.83 (0.39, 1.83)	0.55 (0.29, 1.12)	1.72 (0.70, 4.26)
**24.49 (15.38, 40.49)**	**6.96 (4.86, 10.08)**	1.93 (0.85, 4.15)	Ipilimumab 10 mg/kg	**2.17 (1.68, 2.80)**	1.60 (0.85, 3.09)	1.06 (0.62, 1.86)	**3.31 (1.45, 7.41)**
**11.25 (6.65, 19.84)**	**3.21 (2.06, 4.97)**	0.89 (0.37, 2.00)	**0.46 (0.36, 0.59)**	Ipilimumab 3 mg/kg	0.74 (0.37, 1.49)	**0.49 (0.27, 0.90)**	1.52 (0.65, 3.55)
**15.38 (9.78, 23.67)**	**4.36 (2.04, 9.08)**	1.20 (0.55, 2.54)	0.63 (0.32, 1.18)	1.36 (0.67, 2.70)	Other chemotherapy	**0.66 (0.45, 0.96)**	2.08 (0.89, 4.50)
**23.25 (17.61, 29.80)**	**6.57 (3.36, 12.56)**	1.83 (0.89, 3.47)	0.94 (0.54, 1.61)	**2.05 (1.11, 3.69)**	**1.51 (1.04, 2.21)**	IFN pooled	**3.15 (1.45, 6.20)**
**7.37 (3.95, 14.93)**	2.10 (0.87, 5.15)	0.58 (0.23, 1.44)	**0.30 (0.14, 0.69)**	0.66 (0.28, 1.55)	0.48 (0.22, 1.12)	**0.32 (0.16, 0.69)**	Pembrolizumab

[a]The value in each cell represents the hazard ratio (95% credible interval) for the comparison of the treatment indicated in that row versus the treatment indicated in that column; bolded values are statistically significant at the 0.05 significance level.

IFN: interferon.

Table D.3

Odds ratio estimates[a] from a fixed-effects network meta-analysis of discontinuation in patients with stage II-IV melanoma.

Observation or placebo	1.46 (0.82, 2.56)	**0.35 (0.21, 0.56)**	1.79 (0.92, 3.58)	**0.32 (0.20, 0.53)**	0.97 (0.76, 1.25)
0.68 (0.39, 1.22)	Nivolumab	**0.24 (0.18, 0.31)**	1.23 (0.52, 3.01)	**0.22 (0.11, 0.48)**	0.67 (0.36, 1.25)
**2.89 (1.78, 4.84)**	**4.23 (3.20, 5.61)**	Ipilimumab 10 mg/kg	**5.20 (2.29, 12.20)**	0.93 (0.47, 1.92)	**2.81 (1.64, 4.97)**
0.56 (0.28, 1.09)	0.81 (0.33, 1.94)	**0.19 (0.08, 0.44)**	Other chemotherapy	**0.18 (0.11, 0.29)**	0.54 (0.26, 1.11)
**3.11 (1.88, 5.08)**	**4.54 (2.08, 9.52)**	1.07 (0.52, 2.11)	**5.56 (3.51, 9.02)**	IFN pooled	**3.02 (1.73, 5.22)**
1.03 (0.80, 1.32)	1.50 (0.80, 2.77)	**0.36 (0.20, 0.61)**	1.84 (0.90, 3.82)	**0.33 (0.19, 0.58)**	Pembrolizumab

[a]The value in each cell represents the hazard ratio (95% credible interval) for the comparison of the treatment indicated in that row versus the treatment indicated in that column; bolded values are statistically significant at the 0.05 significance level.
IFN: interferon.
